# FAAP100 is required for the resolution of transcription-replication conflicts in primordial germ cells

**DOI:** 10.1186/s12915-023-01676-1

**Published:** 2023-08-15

**Authors:** Weiwei Xu, Yajuan Yang, Yongze Yu, Canxin Wen, Simin Zhao, Lili Cao, Shidou Zhao, Yingying Qin, Zi-Jiang Chen

**Affiliations:** 1 Center for Reproductive Medicine, Shandong University, Jinan, 250012 Shandong China; 2 State Key Laboratory of Reproductive Medicine and Offspring Health, Shandong University, Jinan, 250012 Shandong China; 3 Key Laboratory of Reproductive Endocrinology of Ministry of Education, Shandong University, Jinan, 250012 Shandong China; 4 Shandong Key Laboratory of Reproductive Medicine, Jinan, 250012 Shandong China; 5 Shandong Provincial Clinical Research Center for Reproductive Health, Jinan, 250012 Shandong China; 6 Shandong Technology Innovation Center for Reproductive Health, Jinan, 250012 Shandong China; 7 National Research Center for Assisted Reproductive Technology and Reproductive Genetics, Shandong University, Jinan, 250012 Shandong China; 8 Research Unit of Gametogenesis and Health of ART-Offspring, Chinese Academy of Medical Sciences (No.2021RU001), Jinan, 250012 Shandong China; 9 Shanghai Key Laboratory for Assisted Reproduction and Reproductive Genetics, Shanghai, 200135 China; 10 Center for Reproductive Medicine, Ren Ji Hospital, School of Medicine, Shanghai Jiao Tong University, Shanghai, 200135 China

**Keywords:** Primordial germ cells, FAAP100, Genome stability, Transcription-replication conflicts, R-loop

## Abstract

**Background:**

The maintenance of genome stability in primordial germ cells (PGCs) is crucial for the faithful transmission of genetic information and the establishment of reproductive reserve. Numerous studies in recent decades have linked the Fanconi anemia (FA) pathway with fertility, particularly PGC development. However, the role of FAAP100, an essential component of the FA core complex, in germ cell development is unexplored.

**Results:**

We find that FAAP100 plays an essential role in R-loop resolution and replication fork protection to counteract transcription-replication conflicts (TRCs) during mouse PGC proliferation. FAAP100 deletion leads to FA pathway inactivation, increases TRCs as well as cotranscriptional R-loops, and contributes to the collapse of replication forks and the generation of DNA damage. Then, the activated p53 signaling pathway triggers PGC proliferation defects, ultimately resulting in insufficient establishment of reproductive reserve in both sexes of mice.

**Conclusions:**

Our findings suggest that FAAP100 is required for the resolution of TRCs in PGCs to safeguard their genome stability.

**Supplementary Information:**

The online version contains supplementary material available at 10.1186/s12915-023-01676-1.

## Background

The genetic information in germ cells is transmitted faithfully between generations to maintain the species continuation. Preserving the genome stability of primordial germ cells (PGCs), which are the progenitors of gametes, is of vital importance to subsequent gametogenesis [1]. The PGC population established by mitotic proliferation during the embryonic stage is a foundation for the reproductive reserve [2]. Previous studies have revealed that genes involved in various DNA damage repair (DDR) pathways, such as *Ercc1, Mcm9, Rad54, Helq* and several Fanconi anemia (FA) genes, are essential for PGC development [3–7]. However, the underlying mechanisms by which these DDR pathways safeguarding PGC development remain largely unknown.

The FA pathway is composed of 22 FA proteins and 5 FA-associated factors and is responsible for interstrand crosslink (ICL) repair [8, 9]. FA gene mutations usually cause FA, which is a rare genetic syndrome characterized by bone marrow failure, somatic malformations, cancer predisposition and reproductive defects [10, 11]. The FA proteins can be classified into three groups according to their roles in ICL repair [12–15]. Group 1 proteins are members of the core complex, including FANCA, FANCB, FANCC, FANCE, FANCF, FANCG, FANCL, FANCM and Fanconi anemia-associated proteins (FAAP10, FAAP16, FAAP20, FAAP24 and FAAP100) [16, 17]. Group 2 comprises the FANCD2-FANCI complex [18]. Group 3 includes the downstream repair factors XPF/FANCQ, SLX4/FANCP, BRCA2/FANCD1, etc. During ICL repair, the FA core complex recognizes the lesion sites and monoubiquitinates the FANCD2-FANCI complex, which then recruits downstream factors to repair lesions by translesion synthesis and homologous recombination [11].

Recently, increasing evidence has indicated that the FA pathway functions in the replication stress response [19–21]. Replication stress is a condition in which the progression of the replication fork (RF) is impeded and the replication rate is slowed [22, 23]. In eukaryotes, the replication machinery may encounter numerous obstacles, such as DNA lesions, repetitive DNA regions, and transcription complexes [23]. Among these obstacles, when replication and transcription proceed simultaneously in the same genomic region, these two complexes inevitably collide due to their shared template; this situation is termed transcription–replication conflict (TRC) [24]. TRC can cause RF stalling and promote the formation of R-loop, a three-stranded structure that is formed when the nascent RNA reanneals to the template DNA strand, displacing the nontemplate strand as single-stranded DNA (ssDNA) [25]. Thus, TRC is an important source of endogenous replication stress and threatens genome stability [24, 26]. Recently, we found that actively proliferating PGCs, coupling with hypertranscription, faced with frequent collisions between transcription and replication, i.e., TRCs. The unique developmental state with high levels of endogenous replication stress imposed by frequent TRCs underlay the increased requirement of the FANCI and FANCD2, the group 2 proteins of the FA pathway, to safeguard genome stability in PGCs [27].

Fanconi anemia core complex-associated protein 100 (FAAP100), a conserved protein in vertebrates, is a key member of the FA core complex [28]. FAAP100 associates with FANCB and FANCL to act as a scaffold for the assembly of the remaining subunits [29, 30] and function as the catalytic module of an ubiquitin ligase [31, 32]. Knockdown of FAAP100 reduced the levels of other components of the FA core complex, including FANCA, FANCB, FANCL, and FANCG [28]. In addition, FAAP100-deficient cells completely lost the ability to ubiquitinate FANCD2 [31, 32], indicating that FAAP100 is essential for the activation of the FA pathway. However, its physiological function in vivo needs to be further explored.

In the present study, we found that *Faap100*^*−/−*^ mice shared a hypogonadism phenotype with FA-null mouse models. Depletion of FAAP100, caused an increase in R-loops and augmented TRCs as well as RF instability, resulting in DNA damage and p53 pathway activation in PGCs. The decrease in PGC number due to proliferation defects ultimately caused insufficient reproductive reserve in *Faap100*^*−/−*^ mice. Our study suggests that FAAP100 protects the PGC from TRC-induced genome instability by resolving R-loops and stabilizing RFs and then ensures the establishment of reproductive reserve.

## Results

### FAAP100 deficiency leads to germ cell loss after birth

We first detected *Faap100* expression in various mouse tissues and found that its expression in testis and oocytes was relatively high (Additional file 1: Fig. S1A). Then, *Faap100*^*−/−*^ mice were generated by using CRISPR/Cas9-mediated genome editing and genotyped (Additional file 1: Fig. S1B, C). To verify that the generated allele of *Faap100* was a null mutation, we detected *Faap100* expression by in situ hybridization using a probe targeting the knockout region of *Faap100.* While *Faap100* mRNAs were detected in testis as well as PGCs and somatic cells of the embryonic day (E) 11.5 genital ridge in the wild-type control, there were no signals in these tissues from *Faap100*^*−/−*^ mice (Additional file 1: Fig. S1D, E).

**Fig. 1:**
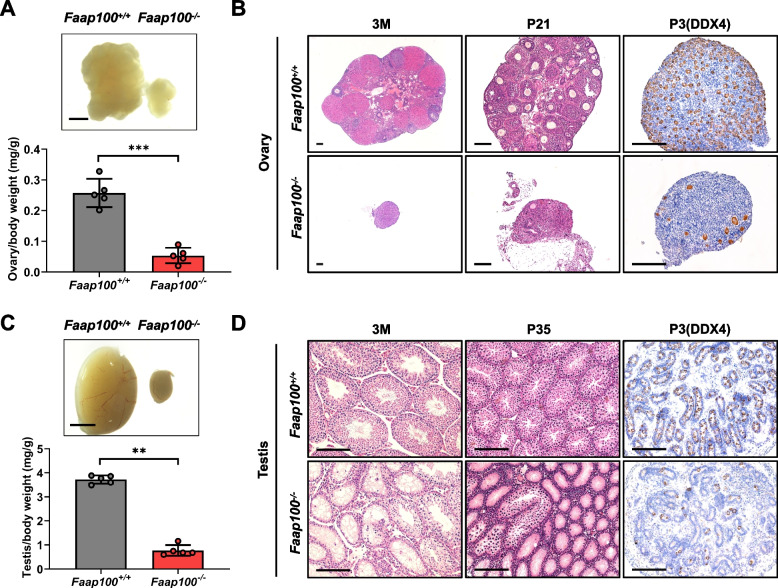
Deletion of *Faap100* causes germ cell loss after birth in both sexes. **A** Gross morphology of ovaries and ovary/body weight ratio of 3-month-old wild-type and *Faap100*^*−/−*^ mice. *n* = 5 per genotype. Scale bars, 500 μm. **B** Haematoxylin & eosin (H&E) and DDX4-stained sections of ovaries at the indicated ages. Scale bars, 100 μm. **C** Gross morphology of testes and testis/body weight ratio of 3-month-old wild-type and *Faap100*^*−/−*^ mice. *n* = 5 per genotype. Scale bars, 2 mm. **D** H&E- and DDX4-stained sections of testes at the indicated ages. Scale bars, 100 μm. Data are shown as the mean ± SD (**A**, **C**). Unpaired two-tailed Student’s *t*-test (**A**) and two-tailed Mann–Whitney *U*-test (**C**), **P < 0.01 and ***P < 0.001

After mating the heterozygous mice, *Faap100*^*−/−*^ mice were born at the expected Mendelian ratio and grew with no obvious abnormalities (Additional file 2: Fig. S2A-C). However, compared to wild-type controls, smaller gonads and reduced gonad weights were observed in both adult *Faap100*^*−/−*^ males and *Faap100*^*−/−*^ females (Fig. 1A, C). Histological analysis of 3-month ovaries showed that abundant follicles at all stages could be observed in wild-type controls, whereas the *Faap100*^*−/−*^ ovaries were atrophic and lacked follicles. The day of the mice born was recorded as postnatal day (PD) 0, and at PD21 as well as PD3, only a few follicles were left in *Faap100*^*−/−*^ ovaries (Fig. 1B). In addition, *Faap100*^*−/−*^ females displayed high levels of serum follicle-stimulating hormone (FSH) and the disappearance of estrous cycles (Additional file 2: Fig. S2D, E), indicating that *Faap100*^*−/−*^ females phenocopied human premature ovarian insufficiency (POI). Meanwhile, we carried out histological analysis on testes from wild-type and *Faap100*^*−/−*^ mice. At 3 months, while spermatogenic cells at all stages were present in wild-type testes, the vast majority of seminiferous tubules in *Faap100*^*−/−*^ testes were devoid of germ cells, and only a few tubules had residual spermatogenic cells. So did the testes of PD35, when the first wave of spermatogenesis was completed [33] (Fig. 1D). Further, there was a significant decrease of spermatogonia in *Faap100*^*−/−*^ testes at PD3 when spermatogonia still did not begin to differentiate [34] (Fig. 1D). Given that both *Faap100*^−/−^ females and males exhibited a severe reduction in germ cells as early as PD3, we speculated that the germ cell defects might have arisen during embryogenesis.

### Loss of PGCs in *Faap100*^*−/−*^ mice during embryogenesis

**Fig. 2:**
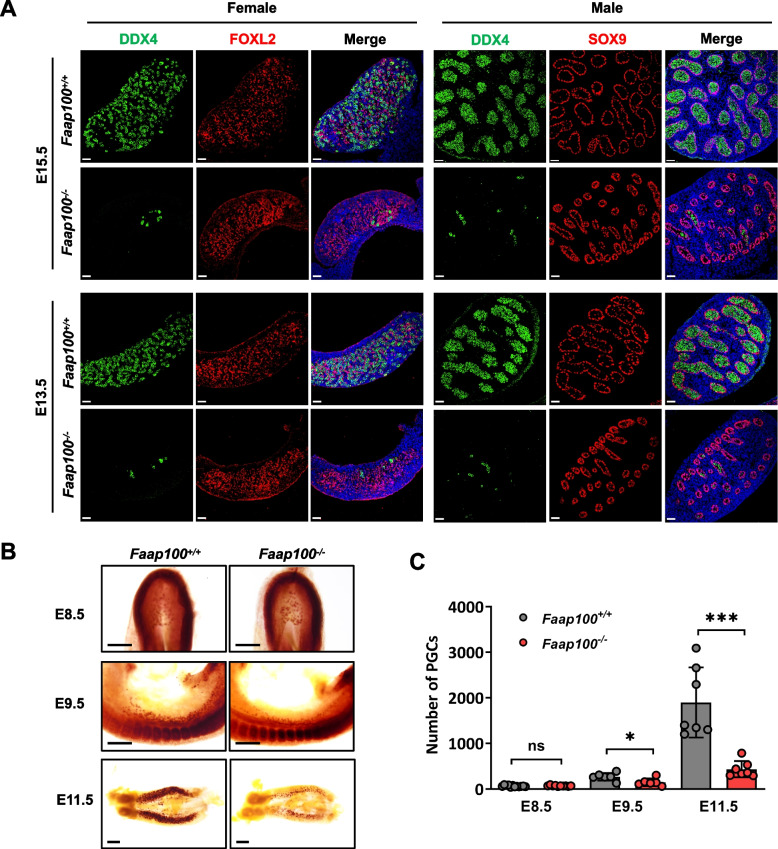
FAAP100 is essential for PGC development. **A** Representative images of E15.5 and E13.5 gonads from wild-type and *Faap100*^*−/−*^ embryos co-immunostained for DDX4 (germ cell marker) and FOXL2 (pre-granulosa marker, in the ovaries) or SOX9 (Sertoli cell marker, in the testis). Scale bars, 50 μm. **B** Representative alkaline phosphatase staining images of wild-type and *Faap100*^*−/−*^ embryos at E8.5 and E9.5 or genital ridges at E11.5. Scale bars, 200 μm. **C** Quantification of PGCs (AP^+^ or STELLA^+^) in wild-type and *Faap100*^*−/−*^ embryos at various embryonic stages as indicated. *n* = 12/10/6/6/7/7 embryos. Data are shown as the mean ± SD (**C**). Unpaired two-tailed Student’s *t*-test (**C**), ns, not significant, *P < 0.05 and ***P < 0.001

To elucidate the consequences of deleting *Faap100* on the development of germ cells and somatic cells in the embryonic gonads, we co-stained cells for the germ cell marker DDX4 and the pregranulosa marker FOXL2 (in ovaries) or the Sertoli cell marker SOX9 (in testes) at E15.5 and E13.5 respectively. While the number of somatic cells in the gonads showed no obvious abnormality, as evidenced by the expression of FOXL2 or SOX9, a dramatically reduced number of DDX4^+^ germ cells was detected in both *Faap100*^*−/−*^ ovaries and testes (Fig. 2A) at E13.5 and E15.5, indicating that *Faap100*^*−/−*^ females and males shared germ cell loss preceding meiosis.

To further determine the onset of germ cell defects, we investigated the earlier PGC pool before meiosis. Alkaline phosphatase staining revealed a significantly reduced number of PGCs in *Faap100*^*−/−*^ embryos compared to wild-type controls at E11.5 and E9.5 (Fig. 2B, C). However, a comparable number of PGCs were found at hindgut epithelium in wild-type and *Faap100*^*−/−*^ embryos at E8.5 (Fig. 2B, C). These results indicated that the establishment of the PGC founder population was normal and the primary defects may have been derived from PGC expansion. Taken together, these data demonstrate that FAAP100 plays a key role in preserving PGC development before meiosis.

### FAAP100 deficiency compromises PGC proliferation

**Fig. 3:**
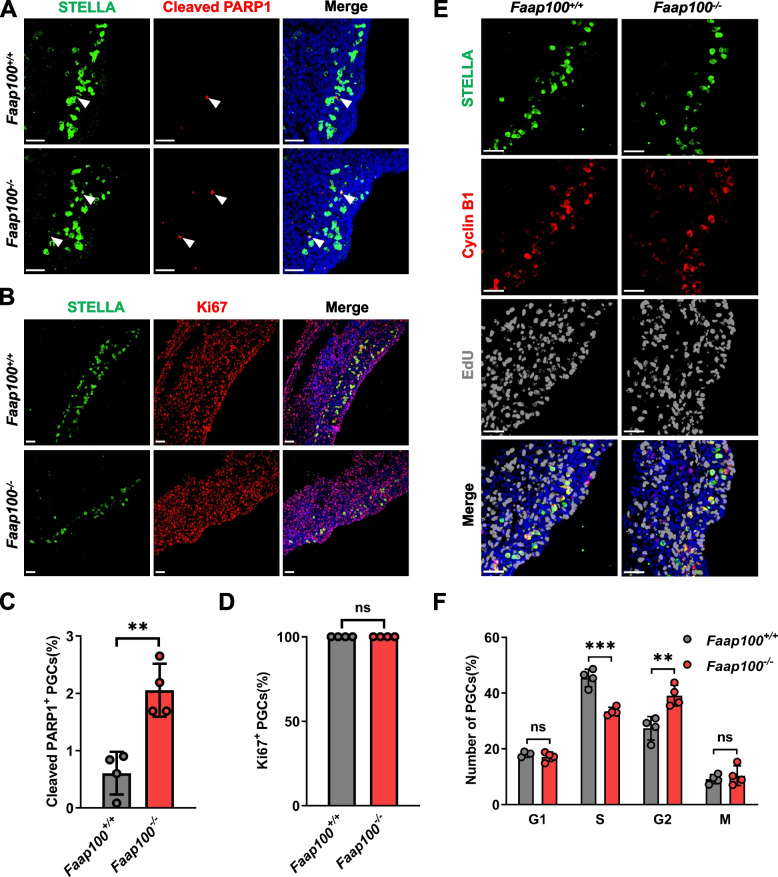
Deletion of *Faap100* results in proliferation defects of PGCs. **A**, **C**, Representative images (**A**) and quantification (**C**) of apoptotic PGCs (STELLA^+^, cleaved PARP1^+^) in *Faap100*^+*/*+^ and *Faap100*^*−/−*^ genital ridges at E11.5. *n* = 4/4 embryos (1067/1000/1344/1200; 365/296/118/339 PGCs). The arrowheads indicate representative apoptotic PGCs. Scale bars, 50 μm. **B**, **D**, Representative images (**B**) and quantification (**D**) of PGCs (STELLA^+^) also positive for Ki67 in *Faap100*^+*/*+^ and *Faap100*^*−/−*^ genital ridges at E11.5. *n* = 4/4 embryos (523/567/636/718; 247/361/249/359 PGCs). Scale bars, 50 μm. **E**, **F**, Representative images (**E**) and quantification (**F**) of the cell cycle distribution of PGCs (STELLA^+^), assessed by co-staining for Cyclin B1 and EdU, in *Faap100*^+*/*+^ and *Faap100*^*−/−*^ genital ridges at E11.5. *n* = 4/4 embryos (1306/656/1046/506; 341/128/337/398 PGCs). In this system, Cyclin B1-negative cells are in G1 phase; EdU-positive cells are in S phase; cells expressing Cyclin B1 in the nucleus are in M phase; and the remaining cells expressing Cyclin B1 in the cytoplasm are in G2 phase. Scale bars, 50 μm. Data are shown as the mean ± SD (**C**, **D**, **F**). Unpaired two-tailed Student’s *t*-test (**C**, **F**) and two-tailed Mann–Whitney *U*-test (**D**), ns, not significant, **P < 0.01 and ***P < 0.001

To determine the reason for PGC defects in the absence of FAAP100, we first assessed the apoptosis rate of E11.5 PGCs using cleaved-PARP1 staining. The results revealed a 3.38-fold increase in the apoptosis rate of *Faap100*^*−/−*^ PGCs compared to that of wild-type controls (2.06 ± 0.46% vs. 0.61 ± 0.37%) (Fig. 3A, C), but the actual counts of apoptotic PGCs were low. We next investigated the proliferation and cell cycle of *Faap100*^*−/−*^ PGCs. We first detected the expression of Ki67, which is expressed in all phases of the cell cycle but not in G0 cells [35]. As in wild-type PGCs, almost all *Faap100*^*−/−*^ PGCs were positive for Ki67 staining, indicating that ablation of FAAP100 did not cause G0 arrest in PGCs (Fig. 3B, D). Then, EdU incorporation combined with Cyclin B1 staining was performed to analyse the cell cycle of PGCs. The results revealed that the percentage of *Faap100*^*−/−*^ PGCs in S phase was reduced compared to that in wild-type controls (33.41 ± 1.47% vs. 45.42 ± 3.15%). In contrast, a 1.43-fold increase in G2 phase cells was observed for *Faap100*^*−/−*^ PGCs compared with wild-type controls (39.07 ± 3.75% vs. 27.35 ± 4.20%). The percentages of *Faap100*^*−/−*^ PGCs in G1 phase and M phase were not significantly different from those in wild-type PGCs (17.15 ± 1.68% vs. 18.11 ± 0.81%; 10.38 ± 3.56% vs. 9.12 ± 1.64%, Fig. 3E, F). Collectively, these data provide evidence that FAAP100 plays a crucial role in safeguarding the rapid proliferation of PGCs.

### FAAP100 deletion did not cause significant abnormalities in PGC migration, transcriptional upregulation and epigenetic reprogramming

After specification, PGCs undergo migration, transcriptional upregulation and epigenetic reprogramming [36], and the disruption of any of these processes will lead to PGC loss and consequently reduced reproductive reserve. To comprehensively determine the impact of FAAP100 deficiency on PGCs, we investigated these developmental events. Alkaline phosphatase staining showed that *Faap100*^*−/−*^ PGCs were embedded in the hindgut epithelium at E8.5, scattered throughout the dorsal mesentery at E9.5, and reached the genital ridge at E11.5. The indistinguishable distribution patterns from wild-type PGCs indicated the normal migration of *Faap100*^*−/−*^ PGCs (Fig. 2B). We next assessed the global transcription levels of PGCs at E11.5 by measuring the expression of RNA Polymerase (Pol) II Ser5 and Ser2 phosphorylation, markers of transcription initiation and elongation, respectively [37, 38]. Immunofluorescence staining showed that *Faap100*^*−/−*^ PGCs exhibited comparable levels of Ser5 and Ser2 phosphorylation of Pol II to those of wild-type controls (Additional file 3: Fig. S3A, B), suggesting that transcriptional upregulation was not obviously affected. Genome-wide epigenetic reprogramming is an essential process in PGC development, and FA proteins have been reported to regulate histone modifications during meiosis [39, 40]. We wondered whether the deletion of FAAP100 perturbed epigenetic reprogramming of PGCs. Hence, histone modifications, including trimethylation of histone H3 at lysine 27 (H3K27me3) and dimethylation of histone H3 at lysine 9 (H3K9me2), and DNA methylation (5mC) in E11.5 PGCs were analysed. Immunofluorescence showed that the majority of PGCs in both *Faap100*^*−/−*^ and wild-type genital ridges erased DNA methylation as well as H3K9me2 modifications and gained high levels of H3K27me3 (Additional file 3: Fig. S3C-E), indicating that there were no obvious abnormalities in epigenetic reprogramming.

### DNA damage accumulation and activation of the p53 signaling pathway in *Faap100*^*−/−*^ PGCs

**Fig. 4:**
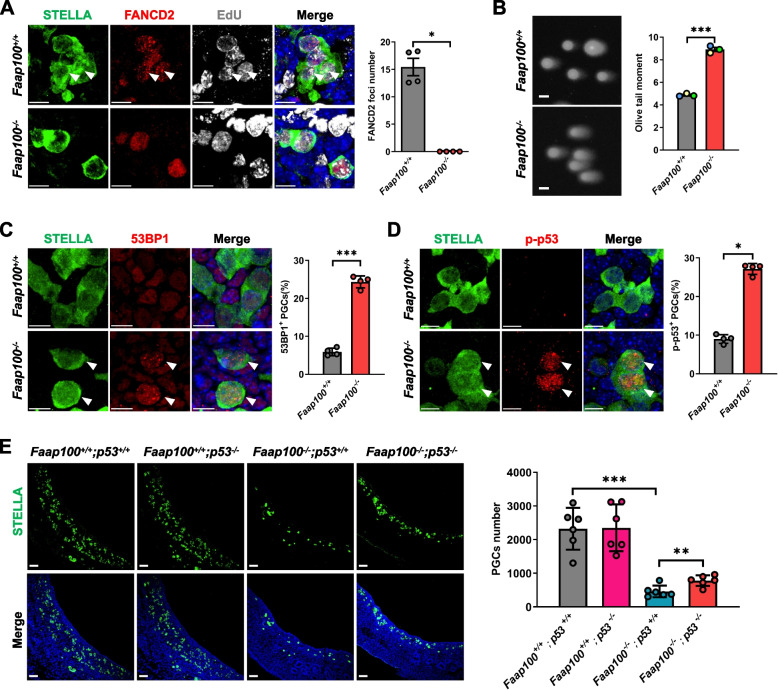
FAAP100 deficiency leads to FA pathway inactivation, DNA damage accumulation and p53 signaling activation in PGCs. **A** Representative images and quantification of FANCD2 foci in PGCs (STELLA^+^) at E11.5. *n* = 4/4 embryos (200/136/145/127; 151/118/140/124 PGCs). The arrowheads indicate FANCD2 foci in PGCs. Scale bars, 10 μm. **B** Representative images and quantification of neutral comet assay tail moment in MACS-sorted PGCs at E11.5. A total of 200 PGCs sorted from 5 embryos of per genotype were scored. Three independent experiments were conducted. Scale bars, 10 μm. **C** Representative images and percentage of PGCs (STELLA^+^) stained positively for 53BP1 in E11.5 genital ridges. *n* = 4/4 embryos (1490/780/786/1340; 362/228/320/299 PGCs). The arrowheads indicate representative 53BP1-positive PGCs. Scale bars, 10 μm. **D** Representative images and percentage of PGCs (STELLA^+^) also positive for p-p53 in E11.5 genital ridges. *n* = 4/4 embryos (522/620/360/352; 311/220/177/226 PGCs). The arrowheads indicate representative p-p53-positive PGCs. Scale bars, 10 μm. **E** Representative images and quantification of PGCs (STELLA^+^) in E11.5 genital ridges of the indicated genotypes. *n* = 6 embryos per genotype. Scale bars, 50 μm. Data are shown as the mean ± SEM (**A**, **B**) or mean ± SD (**C**-**E**). Two-tailed Mann–Whitney *U*-test (**A**, **D**) and unpaired two-tailed Student’s *t*-test (**B**, **C**, **E**), *P < 0.05, **P < 0.01 and ***P < 0.001

Given that FAAP100 is an essential component of the FA core complex, we wondered whether knockout of FAAP100 in vivo inactivated the FA pathway. FANCD2 foci formation or monoubiquitination are biomarkers for FA pathway activation [41]. We first detected FANCD2 foci formation in PGCs. Immunofluorescence analysis revealed that wild-type S-phase PGCs displayed numerous FANCD2 foci, but the foci disappeared in *Faap100*^*−/−*^ PGCs (Fig. 4A). Furthermore, western blotting results showed that the monoubiquitinated form of FANCD2 was observed in wild-type mouse embryonic fibroblasts (MEFs) upon treatment with the ICL inducer mitomycin (MMC) or the replication stress inducer aphidicolin (APH) but not in *Faap100*^*−/−*^ MEFs (Additional file 4: Fig. S4A). These findings verify that FAAP100 is essential for the activation of the FA pathway.

We next assessed DNA damage in *Faap100*^*−/−*^ PGCs by neutral comet assay and found a significant increase in olive tail moment in E11.5 *Faap100*^*−/−*^ PGCs (Fig. 4B). We further found increased proportions of 53BP1 foci-positive *Faap100*^*−/−*^ PGCs (Fig. 4C), suggesting that deletion of FAAP100 led to DSB accumulation in PGCs. Consistently, *Faap100*^*−/−*^ MEFs exhibited an extended olive tail moment and increased proportion of 53BP1 foci-positive cells after treatment with MMC or the replication stress inducers APH and hydroxyurea (HU) (Additional file 4: Fig. S4B-C). In addition, in comparison with wild-type MEFs, *Faap100*^*−/−*^ MEFs contained elevated levels of γH2AX after MMC or APH treatment (Additional file 4: Fig. S4A). Moreover, the percentages of micronucleus-positive *Faap100*^*−/−*^ MEFs were also increased following MMC, APH or HU treatment (Additional file 4: Fig. S4D). These results demonstrate that the loss of FAAP100 leads to the accumulation of DNA damage and increased genome instability.

To reduce the detrimental consequences of DNA damage, p53 is usually activated to regulate genes involved in cell cycle arrest and apoptosis [42]. Previous studies have revealed that a hyperactive p53 signaling pathway contributes to PGC loss in *Fancm* mutant mice, as well as to hematopoietic stem cell (HSC) defects in *Fancd2* mutants [43–45]. Likewise, we observed a significant increase in p-p53 positivity proportion in *Faap100*^*−/−*^ PGCs compared to wild-type controls (Fig. 4D). Consistently, *Faap100*^*−/−*^ MEFs also exhibited elevated expression of p-p53 following MMC or APH treatment (Additional file 4: Fig. S4A). To investigate whether activation of p53 was responsible for PGC deficiency in *Faap100*^*−/−*^ embryos, we crossed *Faap100*^+*/−*^ with *p53*^*+/−*^ mice to obtain double homozygous mutants for both genes. Interestingly, deletion of p53 partially relieved PGC loss in the *Faap100*^*−/−*^ genital ridge at E11.5 (Fig. 4E). These data clarify that the accumulated DNA damage activates p53 signaling pathway in *Faap100*^*−/−*^ PGCs, leading to the profound defects of PGCs.

### FAAP100 counteracts TRCs by R-loop resolution and RF protection

**Fig. 5:**
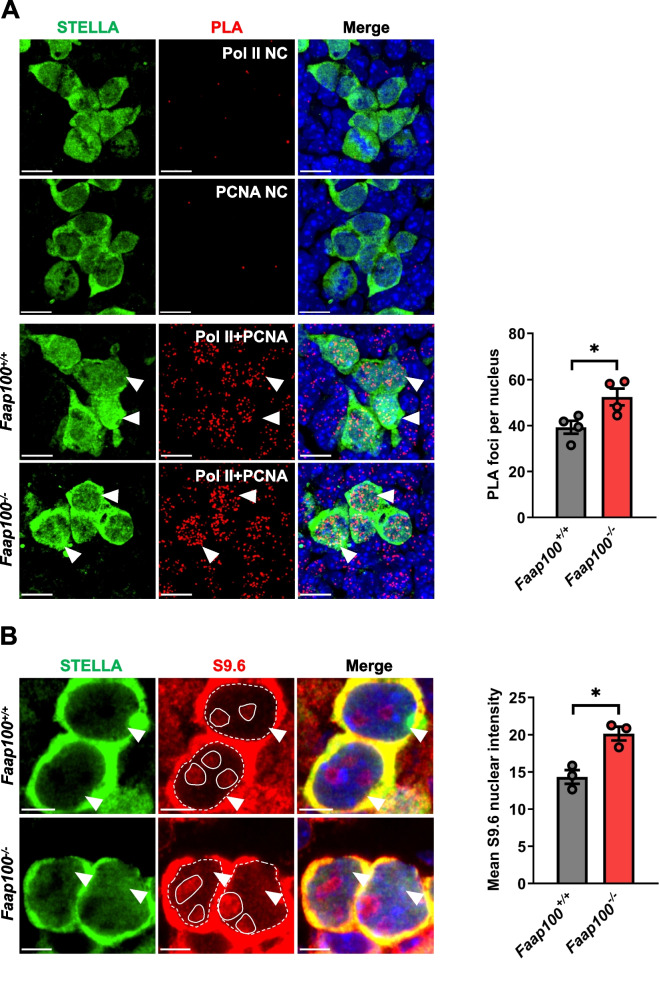
TRCs and R-loops accumulate in *Faap100*^*−/−*^ PGCs. **A** Representative images and quantification of Pol II + PCNA PLA foci in E11.5 PGCs (STELLA^+^). Pol II alone staining and PCNA alone staining were used as single antibody negative control (NC). *n* = 4/4 embryos (202/150/150/156; 208/150/151/150 PGCs). The arrowheads indicate representative PGCs. Scale bars, 10 μm. **B** Representative images and quantification of S9.6 nuclear signal intensity after subtracting the nucleolar signal circled with the solid lines in E11.5 PGCs (STELLA^+^). *n* = 3/3 embryos (207/205/216; 210/197/201 PGCs). The arrowheads indicate representative PGCs. Scale bars, 5 μm. Data are shown as the mean ± SEM (**A**, **B**). Unpaired two-tailed Student’s *t*-test (**A**, **B**), *P < 0.05

**Fig. 6:**
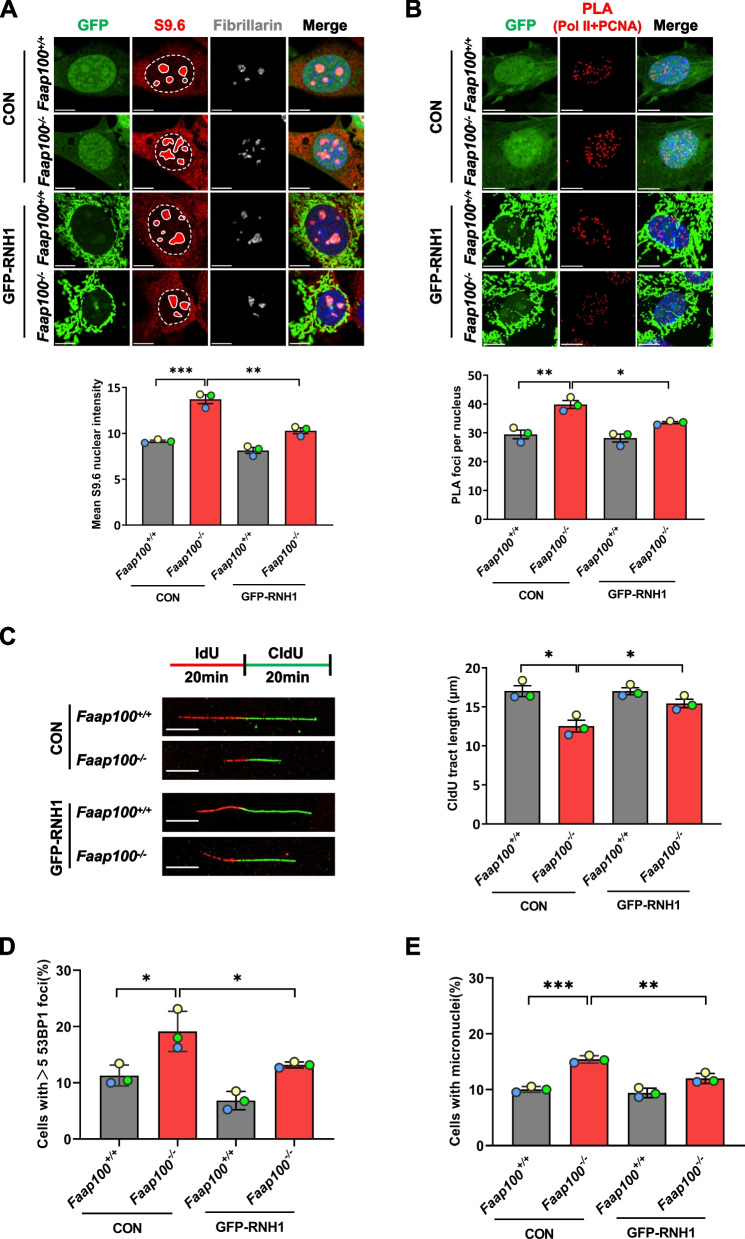
R-loop accumulation aggravates TRCs and impedes fork progression in *Faap100*^*−/−*^ MEFs. **A** Representative images and quantification of S9.6 nuclear signal intensity (exclusive of nucleolar signal that is positive for fibrillarin staining) in MEFs overexpressing control (GFP) or GFP-RNaseH1 (GFP-RNH1) adenovirus. At least 200 cells were scored per group. Three independent experiments were conducted. Scale bars, 10 μm. **B** Representative images and quantification of Pol II + PCNA PLA foci in MEFs treated as in **A**. At least 200 cells were scored per group. Three independent experiments were conducted. Scale bars, 10 μm. **C** Representative images and quantification of CldU tract lengths in MEFs treated as in **A**. At least 200 DNA fibers were scored per group. Three independent experiments were conducted. Scale bars, 10 μm. **D**, **E**, Percentage of MEFs containing > 5 53BP1 foci (**D**) or micronuclei (**E**) after treatment as in **A**. At least 200 cells were scored per group. Three independent experiments were conducted. Data are shown as the mean ± SEM (**A**-**C**) or mean ± SD (**D**, **E**). Unpaired two-tailed Student’s *t*-test (**A**-**E**), *P < 0.05, **P < 0.01 and ***P < 0.001

FAAP100 deletion augments DNA damage in PGCs, whereas the source of endogenous DNA damage remains to be determined. Recently, we found that frequent collisions between transcription and replication, i.e., TRCs existed in actively proliferating PGCs, leading to high levels of endogenous replication stress and activation of the FA pathway [27]. Considering the important role of FAAP100 in the FA pathway, we evaluated the frequency of TRCs with a proximity ligation assay (PLA) in which antibodies against PCNA and RNA Pol II were used to mark replication and transcription machineries, respectively. As indicated by the elevated numbers of PLA foci, FAAP100 depletion significantly aggravated TRCs in both PGCs (Fig. 5A) and MEFs (Fig. 6B).

TRC facilitates the formation of R-loops that further hampers RF progression [25, 46], we therefore evaluated R-loop accumulation in PGCs by staining with the S9.6 antibody, which recognizes DNA:RNA hybrid molecules. The results showed that increased levels of R-loops in *Faap100*^*−/−*^ PGCs (Fig. 5B) and MEFs (Fig. 6A). To further confirm that the accumulated R-loops in *Faap100*^*−/−*^ cells contributed to endogenous DNA damage, we overexpressed RNase H1 (RNH1) in MEFs to remove the RNA moiety of R-loops [47]. As the elevated level of R-loops in *Faap100*^*−/−*^ MEFs was significantly reduced by overexpression of RNH1 (Fig. 6A), TRCs in the absence of FAAP100 were attenuated (Fig. 6B). To better understand the impact of accumulated R-loops on replication progression, we measured RF dynamics directly by the DNA fiber assay. In this assay, MEFs were sequentially pulse-labelled with thymidine analogs IdU and CldU, and the length of CldU track of progressing replication forks with consecutive IdU and CldU signals, was used to evaluate RF velocity. As expected, the shortened CldU tract length in *Faap100*^*−/−*^ MEFs was partially rescued by RNH1 overexpression, suggesting that RF progression was hindered by unscheduled accumulation of R-loops (Fig. 6C). Moreover, the increased genome instability, as indicated by 53BP1 foci and micronucleus formation, in *Faap100*^*−/−*^ MEFs was decreased after overexpression of RNH1 (Fig. 6D, E). These findings suggest that increased R-loops exacerbate TRCs and impede RF progression, leading to DNA damage accumulation in *Faap100*^*−/−*^ cells.

**Fig. 7:**
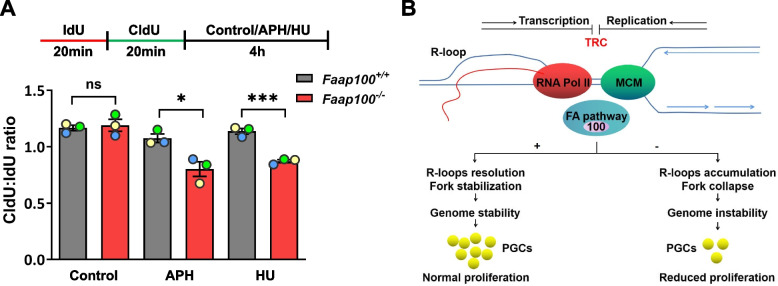
FAAP100 is required to protect nascent DNA strands against degradation. **A** Experimental scheme and ratios of CldU:IdU tract lengths in MEFs under unperturbed conditions or after exposure to 2 μM APH or 0.5 mM HU for 4 h. At least 200 DNA fibers were scored per group. Three independent experiments were conducted. **B** Model of FA pathway functions in the resolution of transcription–replication conflicts (TRCs) in PGCs. When TRCs occur, the FA pathway is activated to resolve R-loops and protect nascent DNA strands from uncontrolled nucleolytic degradation, thus safeguarding PGC genome stability during the active proliferation period. In the absence of FAAP100, the nonfunctional FA pathway leads to R-loop accumulation and replication fork collapse, further resulting in catastrophic genome instability and proliferation defects in PGCs. Data are shown as the mean ± SEM (**A**). Unpaired two-tailed Student’s *t*-test (**A**), ns, not significant, *P < 0.05 and ***P < 0.001

Finally, we determined whether loss of FAAP100 also led to RF instability. Our results showed increased levels of RPA2-S4/8 phosphorylation, which is a well-established marker of DNA end resection [48], following MMC or APH treatment in *Faap100*^*−/−*^ MEFs (Additional file 4: Fig. S4A). To further evaluate the uncontrolled resection in *Faap100*^*−/−*^ MEFs, we performed the DNA fiber assay. In this assay, MEFs were consecutively pulse-labelled with IdU and CldU, and then replication stress inducers APH and HU were used to stall RFs. The nucleolytic resection of nascent DNA (i.e., CldU-labelled DNA) from stalled RFs indicated RF instability. In order to eliminate the influence from the different speed of the RF between groups, CldU/IdU ratio was used to evaluate the resection of the stalled RFs. Under replication stress induced by APH or HU, *Faap100*^*−/−*^ MEFs exhibited decreased CldU:IdU ratios (Fig. 7A), implying the elevated degradation of nascent DNA at stalled forks. Together, these data suggest that FAAP100 counteracts TRCs by resolving R-loops and protecting stalled RFs, thus maintaining the genome stability of PGCs (Fig. 7B).

## Discussion

In this study, we first constructed *Faap100* knockout mouse model and found *Faap100*^*−/−*^ mice had hypogonadism with a dramatic reduction of PGCs from E9.5, a time before entering into meiosis for both male and female germ cells. Further, the number of PGCs in *Faap100*^*−/−*^ embryos reduced to a quarter of wild type embryos at E11.5, which was similar to the phenotype of *Fancd2*^*K559R/K559R*^ mice with FA pathway-dependent function defect, but was less severe than that in *Fancd2*^*−/−*^ mice lacking both FA pathway-dependent and -independent functions [49]. In addition, previous studies reported that *Fanca*^*−/−*^ mice contained half of the number of PGCs in wild type embryos at E11.5 [50]. The differences in the extent of PGC loss may be due to the complete loss of the FA pathway function caused by FAAP100 deletion, whereas disruption of FANCA only partially affect FA pathway activation [31]. Taken together, these data suggest that FAAP100 plays a vital role in PGC development and mainly functions dependently on the FA pathway.

FA pathway inactivation leading to PGC reduction has been observed in FA mouse models for a long time [7]. Based on the function of this pathway in DDR, it is plausible that the accumulation of DNA damage is responsible for PGC reduction. Identifying the sources of endogenous DNA damage in FA-deficient PGCs becomes a major concern in this field and possible sources have been successively uncovered. The classical function of the FA genes is to repair ICL damage, and it has been reported that inactivation of ICL repair caused PGC genome instability in FA-deficient mice [3]. Besides, the de-repression of germline transposable elements was also found to be responsible for the increased DNA damage in PGCs of FA mouse models. Mechanistically, they demonstrated that FANCD2 interacted with PRMT5/piRNA factors to repress the expression of transposable elements [51]. Recently, we found that compared with soma, high frequency of TRCs existed in PGCs, leading to high levels of endogenous replication stress and activation of the FA pathway. The functional defects of FANCI and FANCD2, belonging to the group 2 proteins of the FA pathway, led to the increased TRCs as well as R-loops in PGCs [27]. However, whether the upstream group 1 proteins in the FA pathway are involved in TRC resolution remains to be determined. Here, we first elucidated the role of FAAP100 in vivo and found that it was required for the activation of the FA pathway. Furthermore, we demonstrated that FAAP100 was also essential for the resolution of TRCs in PGCs, and the accumulated DNA damage in FAAP100 deficient cells was derived from TRCs as well as R-loops, reinforcing the role of the FA pathway in this process. Together, our results provide novel insights into the function of FAAP100 in maintaining PGC genome stability and this function may be primarily dependent on activation of the FA pathway.

Given that TRCs seriously threaten genome stability, clarifying the mechanism that resolves these conflicts facilitates our understanding of the biological processes to preserve genetic information. When TRCs occur, the FA pathway is activated to stabilize RFs. Exactly, FANCS/BRCA1, FANCD1/BRCA2, FANCA and FANCD2 were reported to stabilize FANCR/RAD51 on nascent DNA to protect stalled RFs from degradation [20, 52]. Here, we found that FAAP100 was also essential for RF stability. On the other hand, it has been reported that the FA pathway is necessary for R-loop resolution to counteract TRCs [53, 54]. Mechanistically, the MRE11-RAD50-NBS1 (MRN) complex functioned at TRCs to recruit FA proteins to R-loops [55] and the SLX4-RTEL1 complex promoted FANCD2 and RNA Pol II colocalization [56]. FANCI and FANCD2 can directly bind R-loops [57], and FANCD2 can recruit RNA processing enzymes, including hnRNP U and DDX47, to resolve R-loops [58]. Besides, FANCM, which is considered to be epistatic to the FANCI-FANCD2 complex, could also directly remove R-loops through its translocase activity [54]. However, further exploration is needed to determine exactly the mechanism of the FA pathway to resolve R-loops in PGCs.

The FA genes including *Faap100*, are ubiquitously expressed in both germ cells and somatic cells. Several FA-null mouse models showed reproductive defects, growth retardation, skeletal malformations and even embryonic lethality in C57BL/6J background [59], indicating that maintenance of genome stability by the FA pathway was important for both the germ cells and somatic cells. However, the genetic background might be an important determinant for phenotypes of FA-null mice. For example, besides reproductive defects, the FA-null mice with mixed background or other background except C57BL/6J did not present gross developmental defects [59]. Consistently, our *Faap100*^*−/−*^ mice were C57BL/6 and ICR mixed background, and mainly manifested phenotypes of the reproductive system, without embryonic lethality or abnormal development. The reproductive defects always exist regardless of genetic background, suggesting that PGCs are more dependent on the FA pathway to remove unique endogenous genome threats and maintain genome stability. As showed in our previous study, compared with soma, actively proliferating mouse PGCs sustained higher levels of endogenous replication stress imposed by frequent TRCs, which threatened genome stability and activated the FA pathway [27]. Therefore, unique developmental state with a high level of replication stress in PGCs sensitizes them to the functional defects of FA proteins and the phenotype is more obvious in PGCs.

When DNA damage occurs, the DDR pathway is activated to reduce detrimental consequences by halting the cell cycle or inducing apoptosis [60]. Interestingly, because of the absence of the G1-S checkpoint in PGCs [61], they predominantly rely on the G2 checkpoint to safeguard their genome stability. An important cell cycle regulator p53, was reported to be responsible for the elimination of damaged HSCs in FA patients and mice, and deletion of p53 almost completely rescued HSC depletion without changing genomic instability [45, 62]. Inconsistent with this report, although we found that activated p53 prevented the expansion of damaged PGCs, genetic ablation of p53 only partially restored the PGC population. A plausible explanation is that distinct DDR mechanisms may exist in PGCs. Together, in comparison with soma and HSCs, the unique developmental state with high levels of endogenous replication stress and more stringent controls on genome stability may underlie the increased requirement of the DDR pathway in PGCs to maintain the genome stability and restrict the transmission of harmful genetic information to offspring.

While FA patients usually experience fertility defects, mutations of many FA genes, including *FANCM*, *FANCU*, and *FANCA,* also lead to POI in females and non-obstructive azoospermia (NOA) in males [63, 64]. In addition, the FA pathway was also identified as an important genetic determinant of ovarian ageing in a population genetic study [65]. According to our study, the impaired fertility of these patients or accelerated reproductive aging is probably due to the insufficient reproductive reserve caused by the abnormal development of PGCs under TRC-induced replication stress during the foetal stage.

## Conclusions

In summary, our results suggest that FAAP100 protects PGC genome stability against TRCs by resolving R-loops and stabilizing stalled RFs, thereby safeguarding rapid PGC proliferation and the establishment of reproductive reserve. In the absence of FAAP100, the increase in cotranscriptional R-loops exacerbates TRCs and blocks RF progression. Then, the collapse of stalled RFs generates DSBs and consequent genome instability, activating the p53 signaling pathway and resulting in proliferation defects of PGCs and insufficient reproductive reserve (Fig. 7B). This study sheds light on the mechanism of infertility observed in patients with defective FA pathway from the perspective of replication stress-induced PGC abnormality and offers a theoretical basis for the genetic counselling and early intervention of these patients.

## Methods

### Mice

*Faap100*^*−/−*^ mice were obtained by deleting exon 3 to exon 6, and *p53*^*−/−*^ mice were generated by deleting exon 3 to exon 9 using CRISPR/Cas9 technology from Cyagen Bioscience. All mice were maintained in a C57BL/6 J × ICR mixed genetic background and kept under specific pathogen-free conditions with light from 7:00 a.m. to 7:00 p.m. Genotypes of mice were determined by PCR with the primer sequences listed in Additional file 5: Table S1. The euthanesia of mice was performed according to the American veterinary medical association (AVMA) guidelines for the euthanasia of animals (2020). In detail, in order to protect the tissues from being affected by the anesthetic reagent, mice were euthanized by CO_2_ inhalation. All animal procedures were performed according to the guidelines approved by the Animal Care and Use Committee of Shandong University.

### Generation and infection of mouse embryonic fibroblasts (MEFs)

Primary MEFs were obtained from E13.5 embryos and immortalized with SV40 large T antigen lentivirus (Genomeditech) as described previously [66]. In brief, the trunks of mouse embryos were cut into pieces and digested in 0.05% trypsin (Invitrogen) at 37 °C for 4 min with gentle agitation. Then, dissociated cells were collected and cultured in DMEM containing 10% foetal bovine serum and 1% streptomycin/penicillin. After infection with lentivirus expressing SV40 large T antigen for 5 d, MEFs were selected using 0.5 μg/ml puromycin for 3 d to establish the immortalized cell lines.

The overexpression of RNase H1 was conducted by infecting MEFs with RNase H1 adenovirus (Vector Builder) according to the manufacturer’s instructions. The pEGFP empty vectors (Vector Builder) were used as controls. For immunostaining and DNA fiber assays, MEFs were cultured for 72 h after infection with the adenovirus to allow protein expression.

### RNA extraction and quantitative PCR analysis

Total RNA of the desired organs from 21-day-old mice was extracted using TRIzol reagent (Invitrogen) according to standard procedures. The purity and concentration of the RNA were analysed using a NanoDrop ND-1000 spectrophotometer (Thermo Scientific). cDNA was synthesized using a PrimeScript™ RT reagent Kit (TaKaRa). Quantitative PCR was performed with the use of SYBR Green Master Mix (TaKaRa) on a LightCycler 480 system (Roche) following the manufacturer’s procedure. The primers sequences for quantitative PCR are as follows: *Faap100*, forward: GGACGCGAGTTCGTCTATGTG, reverse: ACAGGACGTAGAGTGCCCT; *β-Actin*, forward: AGATGTGGATCAGCAAGCAG, reverse: GCGCAAGTTAGGTTTTGTCA.

### RNA in situ hybridization

The customized RNAscope probe targeting the knockout region of *Faap100* was hybridized on FFPE slides of testis and genital ridges and signals were visualized by using the RNAscope 2.5 HD Reagent Kit-RED (Advanced Cell Diagnostics) according to the manufacturer’s protocol. For counterstaining of PGCs, slides were incubated with STELLA antibody following the standard immunofluorescence protocol after in situ hybridization.

### Haematoxylin & eosin staining

Gonads were fixed in Bouin’s solution overnight at 4 °C and dehydrated in serial concentrations of ethanol. Then, the tissues were embedded in paraffin, sectioned at a thickness of 5 µm, deparaffinized, rehydrated and stained consecutively with haematoxylin and eosin. Images were acquired using an Olympus microscope (BX53).

### Immunohistochemistry

Gonads were fixed with 4% paraformaldehyde overnight at 4 °C, dehydrated, paraffin-embedded, sectioned, deparaffinized, and rehydrated according to standard methods. For antigen retrieval, slides were placed into boiled antigen retrieval buffer (sodium citrate, pH 6.0) for 30 min and cooled to room temperature. Subsequently, slides were immersed in 3% hydrogen peroxide for 15 min at room temperature to quench endogenous peroxidase activity. Then, the slides were blocked and permeabilized in PBS containing 0.3% Triton X-100 and 10% goat serum for 1 h at room temperature and subsequently incubated with primary antibodies (Additional file 6: Table S2) overnight at 4 °C. On the next day, the slides were washed three times in PBS and incubated with a reaction enhancer (Zhongshan Biotech) for 20 min at room temperature. After three washes in PBS, secondary antibody (Zhongshan Biotech) was applied for 20 min at room temperature, followed by three washes in PBS. Thereafter, the slides were incubated with DAB substrate (Vector laboratory). The nuclei were counterstained with haematoxylin. Images were captured with an Olympus microscope (BX53).

### Immunofluorescence

For immunofluorescence of genital ridges, embryos were fixed in 4% paraformaldehyde overnight at 4 °C. The genital ridges were separated and embedded in optimal cutting temperature compound and then serially sectioned into 10 μm slices and mounted on slides. After being washed with PBS for 15 min, the slides were incubated in solution containing 0.3% Triton X-100 and 25% donkey serum for 1 h at room temperature to permeabilize the cells and block nonspecific binding. Then, the samples were incubated with primary antibodies (Additional file 6: Table S2) diluted in 1% bovine serum albumin containing 0.3% Triton X-100 at 4 °C overnight. Slides were washed three times with PBS containing 0.1% Triton X-100 (PBST) and incubated with appropriate secondary antibodies for 1 h at room temperature. Finally, the nuclei were counterstained with Hoechst 33,342, and the slides were mounted. Images were acquired using a confocal microscope (Andor Technology).

For immunofluorescence of 5mC, DNA denaturation was performed by incubation in 2N HCl for 10 min at 37 °C and terminated using 0.1 M sodium borate before blocking. For immunofluorescence of cultured cells, fixation was performed for 15 min at room temperature. The remaining procedures were performed as described above.

### Estrous cycle analysis

Vaginal smears of 8-week-old female mice were taken once a day for 2 weeks. Briefly, to collect cells from the vaginal, the pipette containing 20 µl saline was gently inserted into the margin of vaginal orifice and the saline was flushed into the vagina and back out for 3 times. Then the collected sample was placed evenly on the slide in a thin layer for airdry. Next, the vaginal smears were observed using a microscope after being fixed with 95% alcohol and stained with the haematoxylin & eosin. The different estrous cycle stage (proestrus, estrus, metestrus, diestrus) were classified according to the criteria previously described [67]. Exactly, in proestrus, the predominant feature is the presence of nucleated epithelial cells in the smear. The estrus stage is characterized by the appearance of predominately anucleated keratinized epithelial cells in the smear. In metestrus, the smear is composed of anucleated keratinized epithelial cells and neutrophils. The diestrus stage is characterized by the combination of neutrophils, nucleated epithelial cells and few anucleated keratinized cells.

### Alkaline phosphatase staining

Embryos were fixed in 4% paraformaldehyde for 1 h at 4 °C and washed with PBS three times, followed by two washes for 10 min with 25 mM Tris-maleic acid buffer (pH 9.0). Then, the fixed tissues were stained with freshly prepared staining solution containing 25 mM Tris-maleic acid, 0.5 mM MgCl_2_, 0.4 mg/ml 1-naphthyl phosphate disodium salt and 1 mg/ml Fast Red TR salt for 20–30 min at 37 °C with gentle agitation in a dark incubator shaker. PBS was added to stop the reaction. Subsequently, tissues were washed in ddH_2_O three times and cleared in 40% and 80% glycerol for 1 h at 4 °C. Images were captured using a stereoscope (Nikon).

### Cell cycle analysis with EdU incorporation

E11.5 pregnant mice were intraperitoneally injected with 5-ethynyl-2′-deoxyuridine (EdU) at a single dose of 50 mg/kg and sacrificed 2 h later. Genital ridges were prepared as mentioned above. The detection of EdU was carried out using an EdU Kit (RiboBio) according to the manufacturer’s instructions. In brief, slides were immersed in glycine for 10 min and permeabilized with 0.5% Triton X-100 in PBS for 10 min at room temperature. After two washes with PBS, the slides were incubated with EdU reaction solution for 30 min at room temperature in the dark. Then, the slides were washed three times for 10 min with 0.5% Triton X-100 in PBS. Subsequently, staining for STELLA and Cyclin B1 was carried out following the immunofluorescence protocol described above.

### Magnetic PGC sorting

*Faap100*^*+/−*^ mice were mated, and the pregnant mice were sacrificed at E11.5. Genital ridges collected from embryos of the desired genotypes were placed into one tube containing prewarmed Accutase (Thermo Fisher). Then, the genital ridges were digested at 37 °C for 25 min with gentle agitation, and the digestion was terminated using Leibovitz's L-15 medium supplemented with 10% FBS. After being filtered through a 70 μm SmartStrainers (Miltenyi Biotech), the cells were centrifuged at 300 × g for 8 min at 4 °C and resuspended in 80 μl DPBS containing 0.5% BSA and 2 mM EDTA (buffer). Then, 20 μl anti-SSEA-1 microbeads were added to the cell suspension and incubated for 15 min at 4 °C. Subsequently, the cells were washed, resuspended in buffer and transferred to a prerinsed separation column placed on the magnetic separator. After being washed three times, the column was removed, added with 500 μl buffer and flushed through by a plunger to expel SSEA1-positive cells (PGCs) into the collection tube. Finally, the sorted cells were centrifuged, resuspended and used for the neutral comet assay.

### Neutral comet assay

A neutral comet assay was carried out according to the procedure [68]. In brief, MACS-sorted PGC or MEF suspensions were mixed with molten low-melting-point agarose (Sangon Biotech) and transferred onto prepared slides covered with molten agarose (Sangon Biotech). Samples were lysed in neutral lysis solution (2.5 M NaCl, 100 mM Na_2_EDTA, 10 mM Tris, 1% N-lauroylsarcosine, and 1% Triton X-100 [pH 9.5]) for 1 h at room temperature and unwound in neutral electrophoresis solution (300 mM sodium acetate, 100 mM Tris [pH 8.3]) for 20 min at room temperature. Subsequently, electrophoresis was performed for 20 min at 20 V and 80 mA. After being fixed with 100% ethanol and air-dried, the samples were stained with Hoechst 33,342. Slides were imaged by a fluorescence microscope (Olympus BX53), and CASP comet assay analysis software (Andor Technology) was used to calculate the olive tail moments.

### Western blot

Cells were lysed using 1% SDS buffer containing protease and phosphatase inhibitor (Bimake). Protein concentrations were quantified using a BCA protein assay kit (Thermo Scientific). Equivalent proteins were separated by SDS–PAGE and transferred to polyvinylidene fluoride membranes (Millipore). The membranes were incubated with 5% skim milk for 1 h at room temperature to block nonspecific binding, followed by incubation with primary antibodies (Additional file 6: Table S2) at 4 °C overnight. The next day, the membranes were incubated with the corresponding horseradish peroxidase (HRP)-conjugated secondary antibodies (Proteintech Group) for 1 h at room temperature. Then, signals were detected using an ECL chemiluminescence kit (Millipore) and captured with the ChemiDoc MP Imaging System (Bio-Rad).

### Proximity ligation assay (PLA)

PLA was performed using Duolink PLA Technology (Merck). First, tissues were fixed, dehydrated, embedded, sectioned, deparaffinized, rehydrated, retrieved, permeabilized, blocked, and incubated with primary antibodies (Additional file 6: Table S2) as described for immunohistochemistry analyses. Then, the subsequent proximal ligation assay steps, including PLA probe incubation, ligation and amplification reactions, were carried out according to the manufacturers’ guidelines. Next, immunofluorescence staining of STELLA was performed. Finally, cell nuclei were counterstained with Hoechst 33,342. Images were acquired with a confocal microscope (Andor Technology). For PLA in MEFs, cells were fixed in 4% paraformaldehyde at room temperature for 15 min, permeabilized in cold methanol at -20 °C for 10 min, blocked and incubated with antibody as described for the immunofluorescence analyses. Then, the PLA procedure was performed as described above.

### DNA fiber assay

The DNA fiber assay was performed as described previously [69, 70]. Briefly, MEFs were consecutively pulse-labelled with 25 μM IdU (Sigma) and 250 μM CldU (Sigma) for the indicated time. Then, the cells were digested, collected with ice-cold PBS and lysed on slides, followed by stretching of the DNA fibers. After being fixed with methanol:acetic acid (volume ratio 3:1) for 10 min, DNA fibers were denatured using 2 N HCl for 14 min at 37 °C and blocked with 1% BSA in PBS for 1 h. Subsequently, slides were incubated with primary antibodies (Additional file 6: Table S2) and secondary antibodies as described in the immunofluorescence section. DNA fibers were imaged on a confocal microscope, and ImageJ software was used to measure the DNA tract length. For the experiment with replication stress inducer treatment, cells were exposed to APH (Sigma) or HU (Sigma) for 4 h just after CldU labelling.

### Statistical analysis

All the experiments were repeated at least three independent times. Generally, 100–1500 PGCs per embryo or 3–12 embryos were included to perform statistical analysis. All statistical analyses were carried out using SPSS 21.0 software (IBM) and GraphPad Prism 8. Data from independent experiments with repeated values were presented as the mean ± SEM or mean ± SD. The unpaired two-tailed Student’s *t*-test was used to determine statistical significance for data normally distributed, and the two-tailed Mann–Whitney *U*-test was applied when the data were not normally distributed. Chi-square test was used for analysis of genotype proportion. The difference was considered to be statistically significant when *P* < 0.05.

### Supplementary Information

**Additional file 1: Fig. S1.** Generation and verification of *Faap100* knockout mice. A, Analysis of *Faap100* expression using qRT-PCR in the indicated tissues and cells of wild-type mice at 21 days (P21). The expression of *Actin* was also measured for normalization. Data are shown as the mean ± SEM, *n*=3 mice. B, Top: Schematic diagram showing the strategy for the generation of *Faap100* knockout mice. The scissors indicate the targeting gRNA regions, and black arrows illustrate the primers for genotyping. Bottom: Scheme of the FAAP100 domain structure. Deletion of exons 3-6 led to frameshift and generated a truncated protein. C, Genotyping results of *Faap100* mice. D, E Representative in situ hybridization (RNAscope) images for *Faap100* (red spots) in the testis and genital ridge (D), followed by STELLA immunostaining to mark the PGCs (E). Scale bars, 50 μm.

**Additional file 2: Fig. S2.** Deletion of *Faap100* results in premature ovarian insufficiency. A, Statistical analysis for genotyping results of pups resulting from the mating of heterozygous female mice with heterozygous male mice. *n*=218 pups. B, A representative photograph of 3-month-old wild-type and *Faap100*^*-/-*^ female mice. C, Body weights of 3-month-old wild-type and *Faap100*^*-/-*^ mice. *n*=11/11/8/8. D, Levels of FSH and E2 in the serum of 3-month-old wild-type and *Faap100*^*-/-*^ female mice. FSH, *n*=9/8; E2, *n*=7/7. E, Estrous cycles of 3-month-old wild-type and *Faap100*^*-/-*^ female mice. *n*=6/6. Representative data from 3 independent mice per genotype. E, estrus; P, proestrus; D, diestrus; M, metestrus. Data are shown as the mean ± SD (C, D). Chi-square test (A) and unpaired two-tailed Student’s *t*-test (C, D), ns, not significant and **P < 0.01.

**Additional file 3: Fig. S3.** No significant abnormalities of transcriptional activation and epigenetic modification were observed in *Faap100*^*-/-*^ PGCs. A, Representative images and quantification of pSer5 Pol II signal intensity in E11.5 PGCs (STELLA^+^). *n*= 3/3 embryos (171/208/178; 150/172/150 PGCs). Scale bars, 10 μm. B, Representative images and quantification of pSer2 Pol II signal intensity in E11.5 PGCs (STELLA^+^). *n*= 3/3 embryos (203/161/185; 161/191/152 PGCs). Scale bars, 10 μm. C, Representative images and percentage of PGCs (STELLA^+^) stained negative for 5mC in E11.5 genital ridges. *n*= 3/3 embryos (201/214/206; 207/207/213 PGCs). Scale bars, 10 μm. D, Representative images and percentage of PGCs (STELLA^+^) stained negative for H3K9me2 in E11.5 genital ridges. *n*= 3/3 embryos (236/250/153; 145/140/160 PGCs). Scale bars, 10 μm. E, Representative images and percentage of PGCs (STELLA^+^) stained positive for H3K27me3 in E11.5 genital ridges. *n*= 3/3 embryos (476/359/200; 296/104/111 PGCs). Scale bars, 10 μm. The arrowheads in A-E indicate representative PGCs. Data are shown as the mean ± SEM (A, B) or mean ± SD (C-E). Unpaired two-tailed Student’s *t*-test (A-D) and two-tailed Mann-Whitney *U*-test (E). ns, not significant.

**Additional file 4: Fig. S4.** FAAP100 loss causes elevated genome instability in MEFs. A, Representative images and quantification of the indicated proteins expression in whole-cell MEF extracts after exposure to 50 ng/ml MMC or 0.5 μM APH for 24 h. Three independent experiments were conducted. B, Quantification of the neutral comet assay tail moment in MEFs following exposure to 2 μM APH or 0.5 mM HU for 4 h. At least 200 cells were scored per group. Three independent experiments were conducted. C, Representative images and percentage of MEFs containing > 5 53BP1 foci following 100 ng/ml MMC or 2 μM APH treatment for 4 h. At least 200 cells were scored per group. Three independent experiments were conducted. Scale bars, 10 μm. D, Representative images and percentage of MEFs harbouring micronuclei after exposure to 50 ng/ml MMC, 0.5 μM APH or 0.5 mM HU for 24 h. At least 200 cells were scored per group. Three independent experiments were conducted. The arrowheads indicate micronuclei. Scale bars, 10 μm. Data are shown as the mean ± SD (A, C, D) or mean ± SEM (B). Unpaired two-tailed Student’s *t*-test (A-D), ns, not significant, *P < 0.05, **P < 0.01 and ***P < 0.001.

**Additional file 5: Table S1.** Sequences of PCR primers used for genotyping.

**Additional file 6: Table S2.** Antibodies.

**Additional file 7: Table S3.** Reagents for assays.

**Additional file 8.** Uncropped gels/blots.

**Additional file 9.** Individual data values.

## Data Availability

All data generated or analysed during this study are included in this published article and its supplementary information files. The uncropped gels/blots are provided in Additional file 8. The individual data values for Figs. 1, 2, 3, 4, 5, 6, and 7, as well as Additional file 1: Fig. S1, Additional file 2: Fig. S2, Additional file 3: Fig. S3 and Additional file 4: Fig. S4, are provided in Additional file 9.

## Abbreviations

PGCs: Primordial germ cells
DDR: DNA damage repair
FA: Fanconi anemia
ICL: Interstrand crosslink
RF: Replication fork
TRC: Transcription–replication conflict
ssDNA: Single-stranded DNA
FAAP100: Fanconi anemia core complex-associated protein 100
PD: Postnatal day
MEFs: Mouse embryonic fibroblasts
MMC: Mitomycin
APH: Aphidicolin
HSC: Hematopoietic stem cell
PLA: Proximity ligation assay
RNH1: RNase H1
MRN: MRE11-RAD50-NBS1
EdU: 5-Ethynyl-2′-deoxyuridine
FSH: Follicle-stimulating hormone
POI: Premature ovarian insufficiency
HU: Hydroxyurea
NOA: Non-obstructive azoospermia
AVMA: American veterinary medical association
