# Supplementary figures and images for: FAAP100 is required for the resolution of transcription-replication conflicts in primordial germ cells
Source: BMC Biol. 2023 Aug 15;21:174. doi: 10.1186/s12915-023-01676-1 (PMC10426154; doi:10.1186/s12915-023-01676-1)

**A**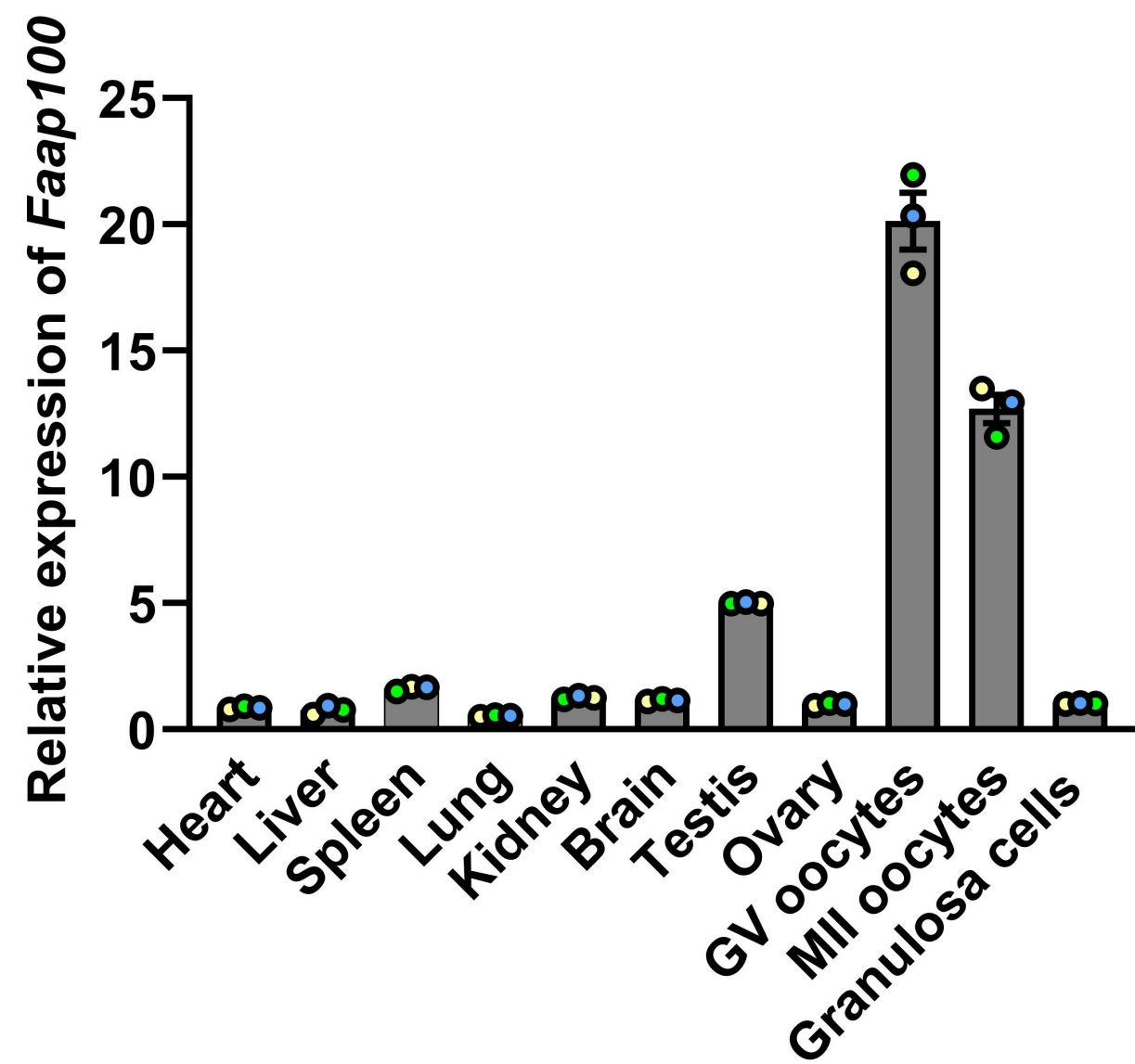**B**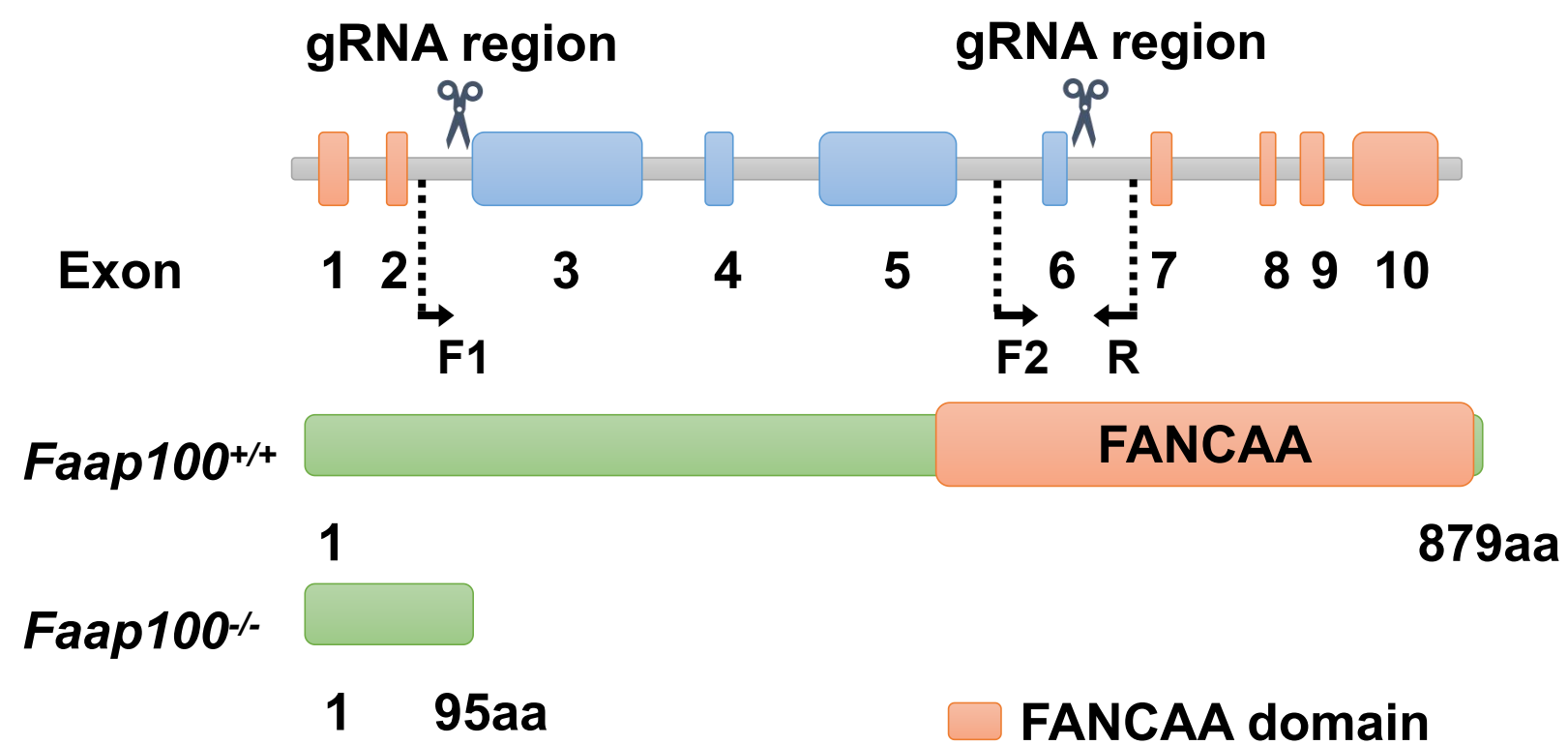**C**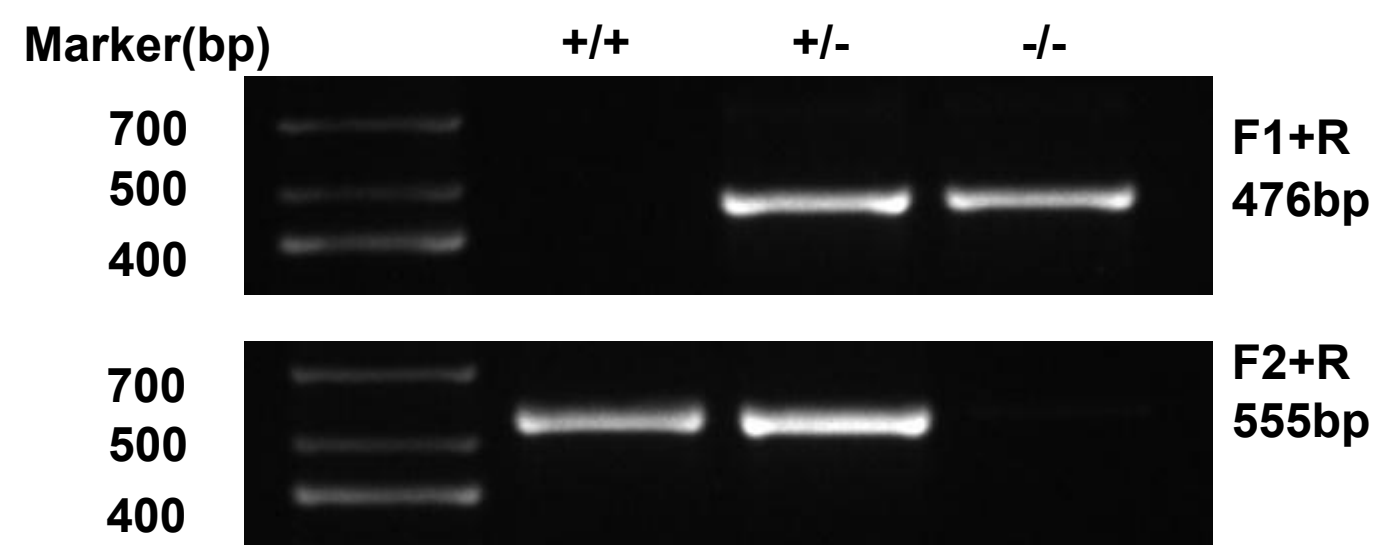**D**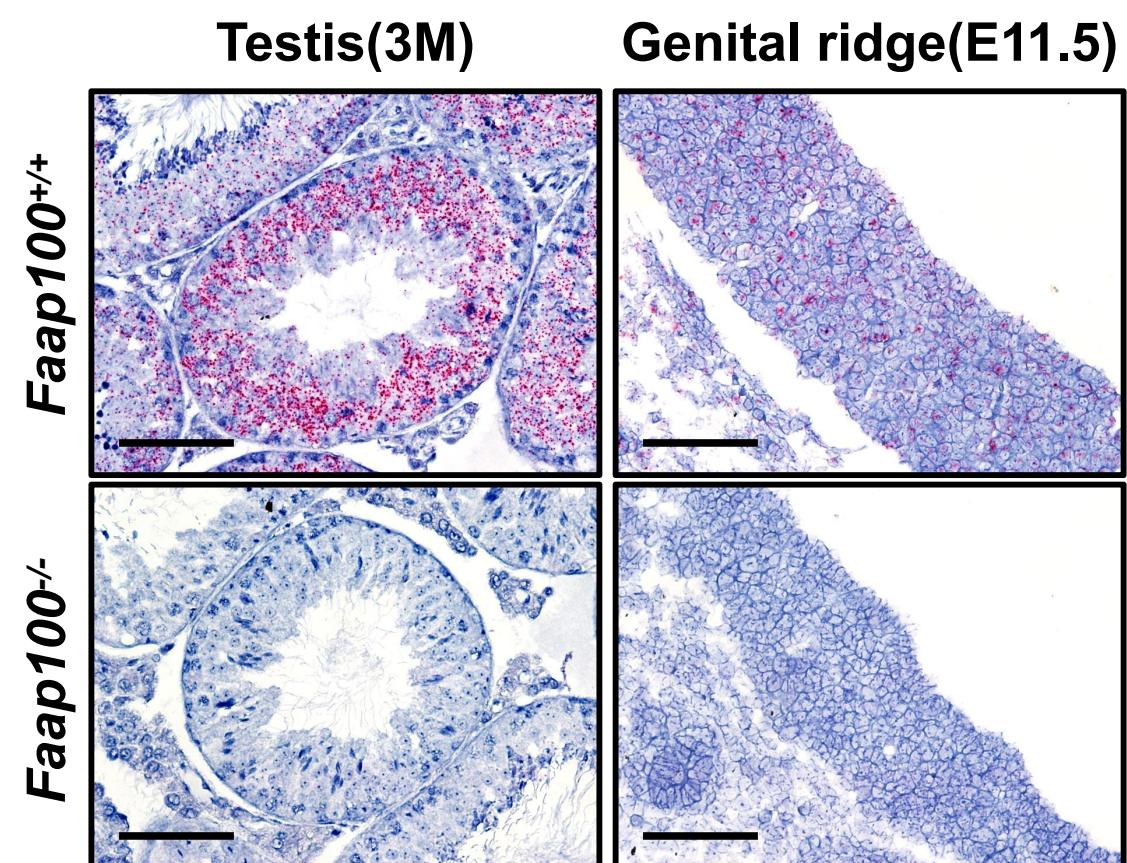**E**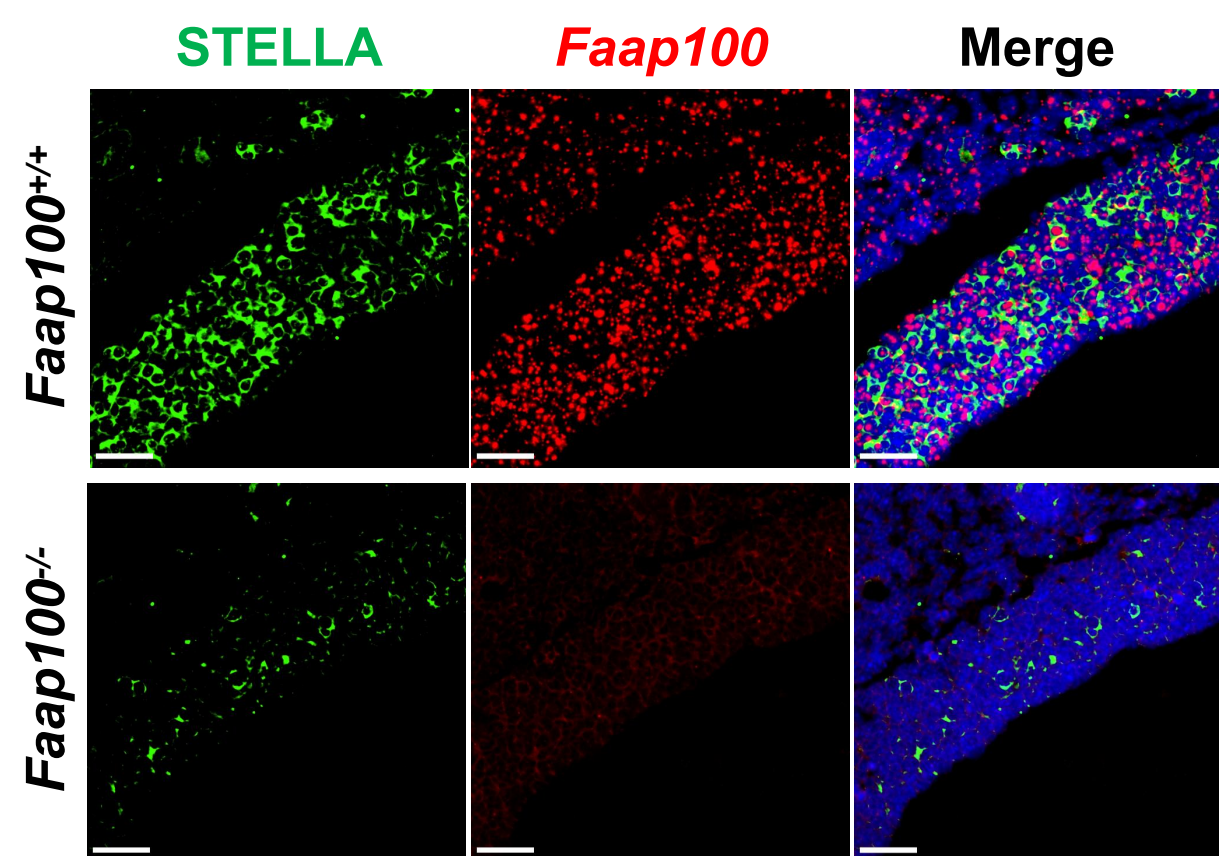

Supplement: Supplementary file 1 — Additional file 1: Fig. S1. Generation and verification of Faap100 knockout mice. A, Analysis of Faap100 expression using qRT-PCR in the indicated tissues and cells of wild-type mice at 21 days (P21). The expression of Actin was also measured for normalization. Data are shown as the mean ± SEM, n=3 mice. B, Top: Schematic diagram showing the strategy for the generation of Faap100 knockout mice. The scissors indicate the targeting gRNA regions, and black arrows illustrate the primers for genotyping. Bottom: Scheme of the FAAP100 domain structure. Deletion of exons 3-6 led to frameshift and generated a truncated protein. C, Genotyping results of Faap100 mice. D, E Representative in situ hybridization (RNAscope) images for Faap100 (red spots) in the testis and genital ridge (D), followed by STELLA immunostaining to mark the PGCs (E). Scale bars, 50 μm. [file 12915_2023_1676_MOESM1_ESM.pdf]

**A**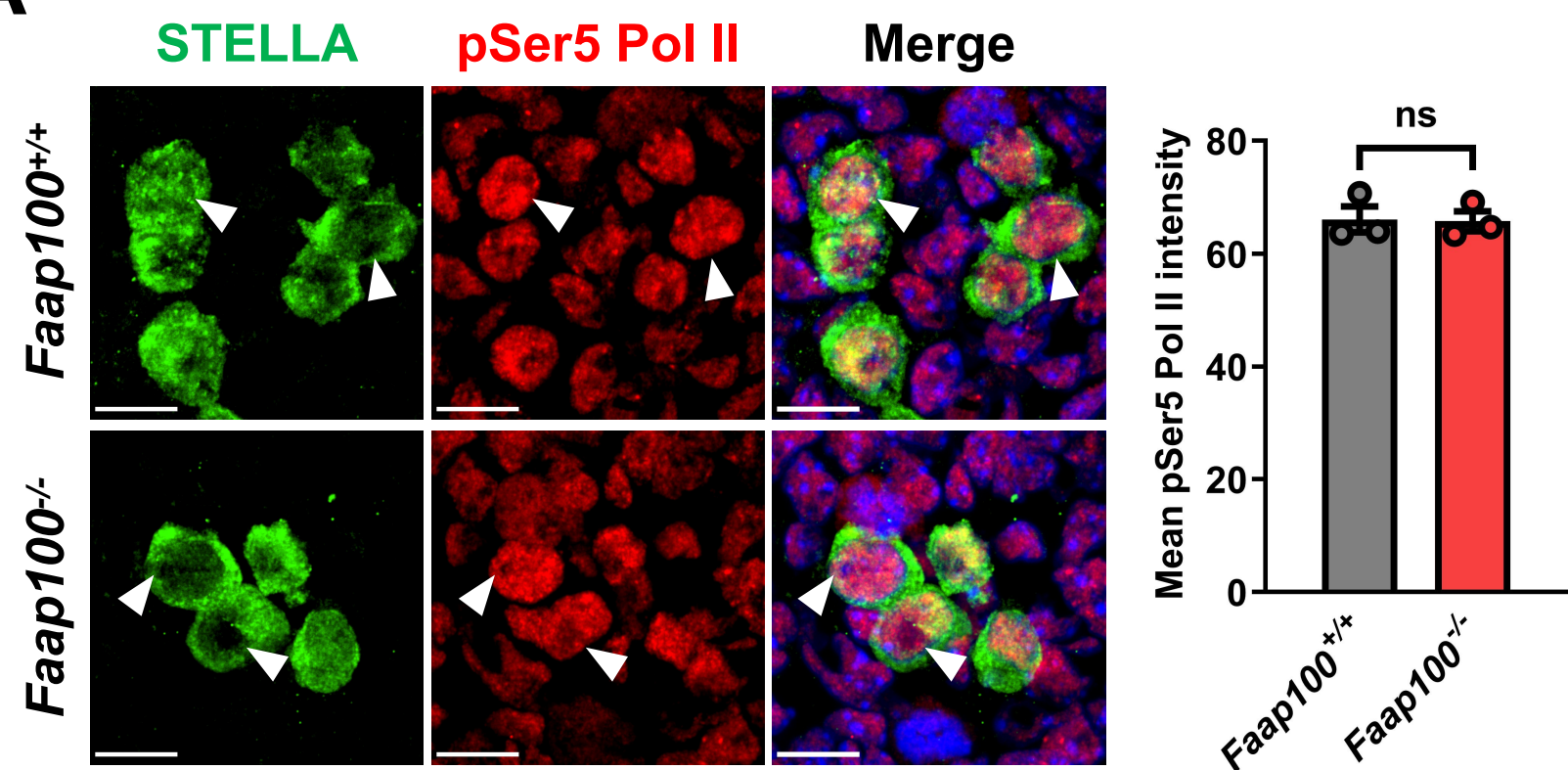**B**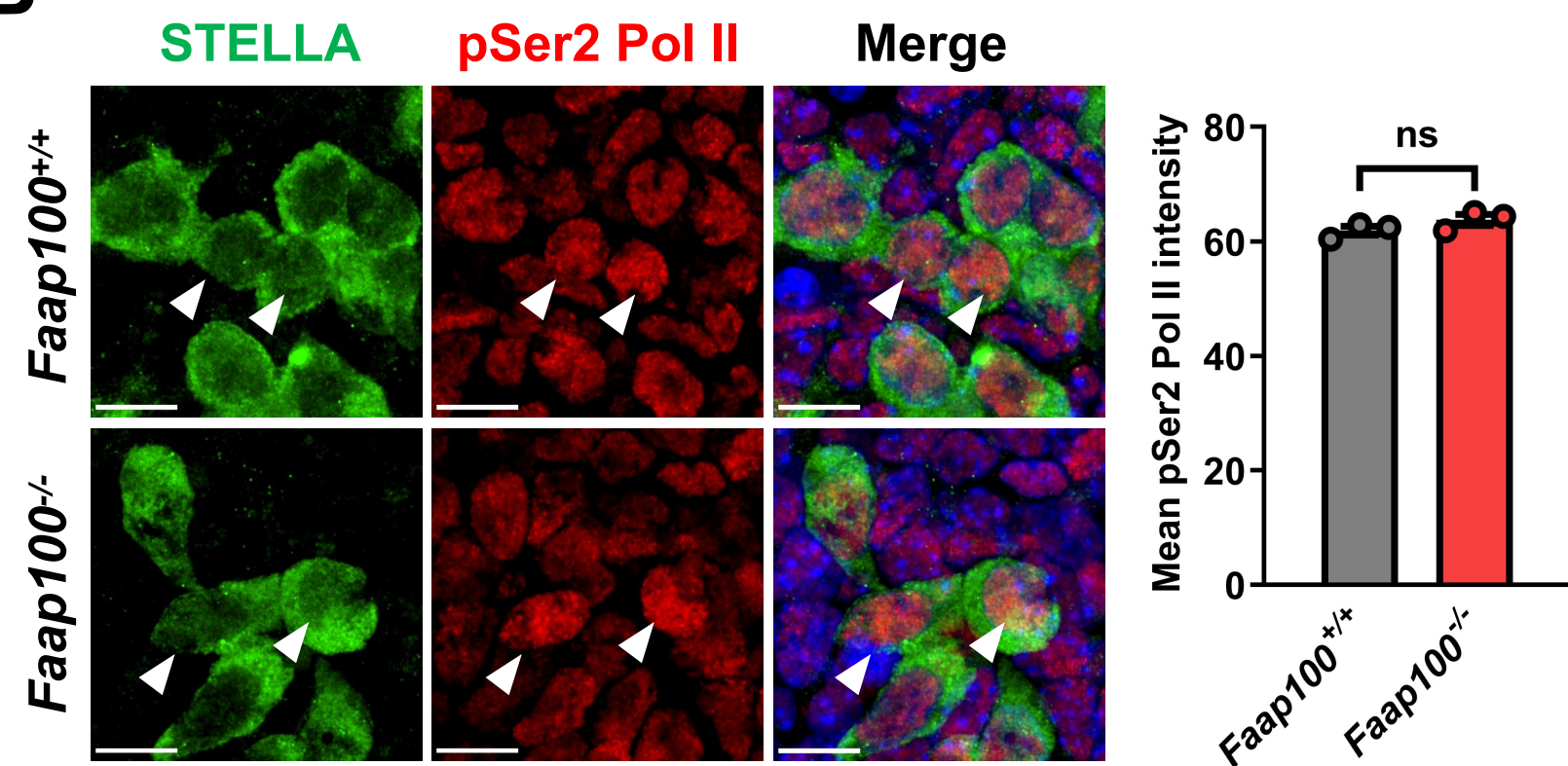**C**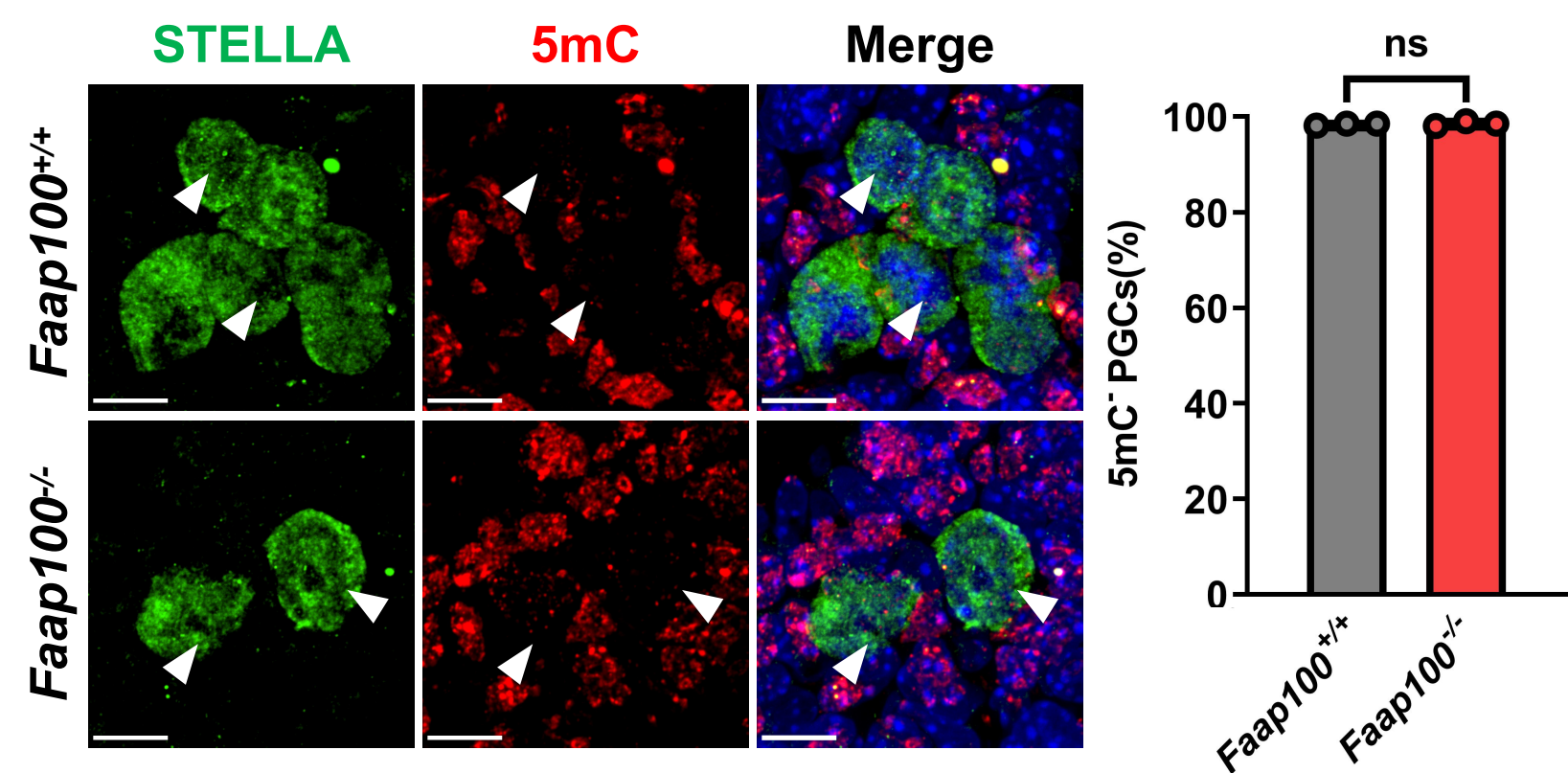**D**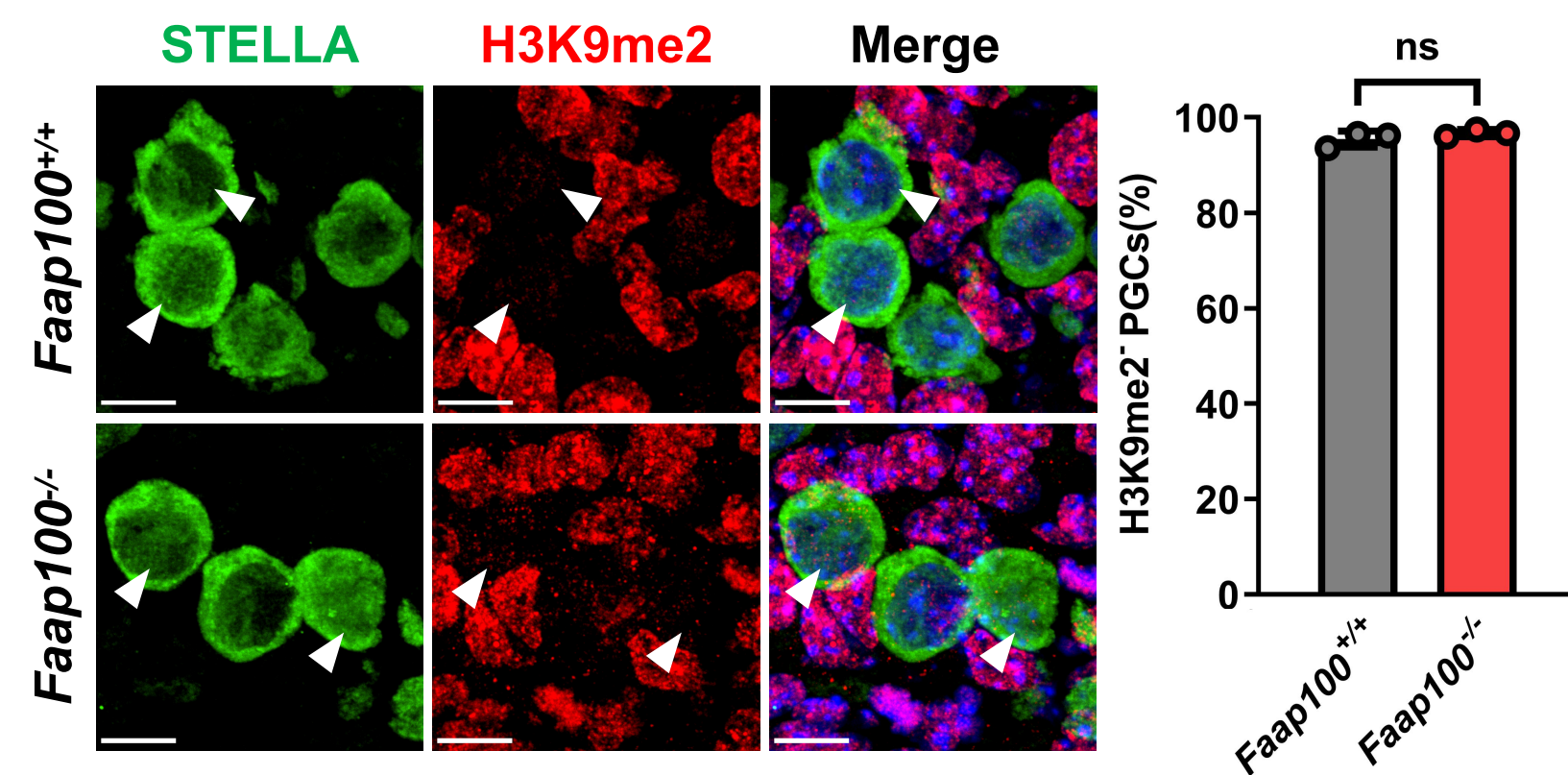**E**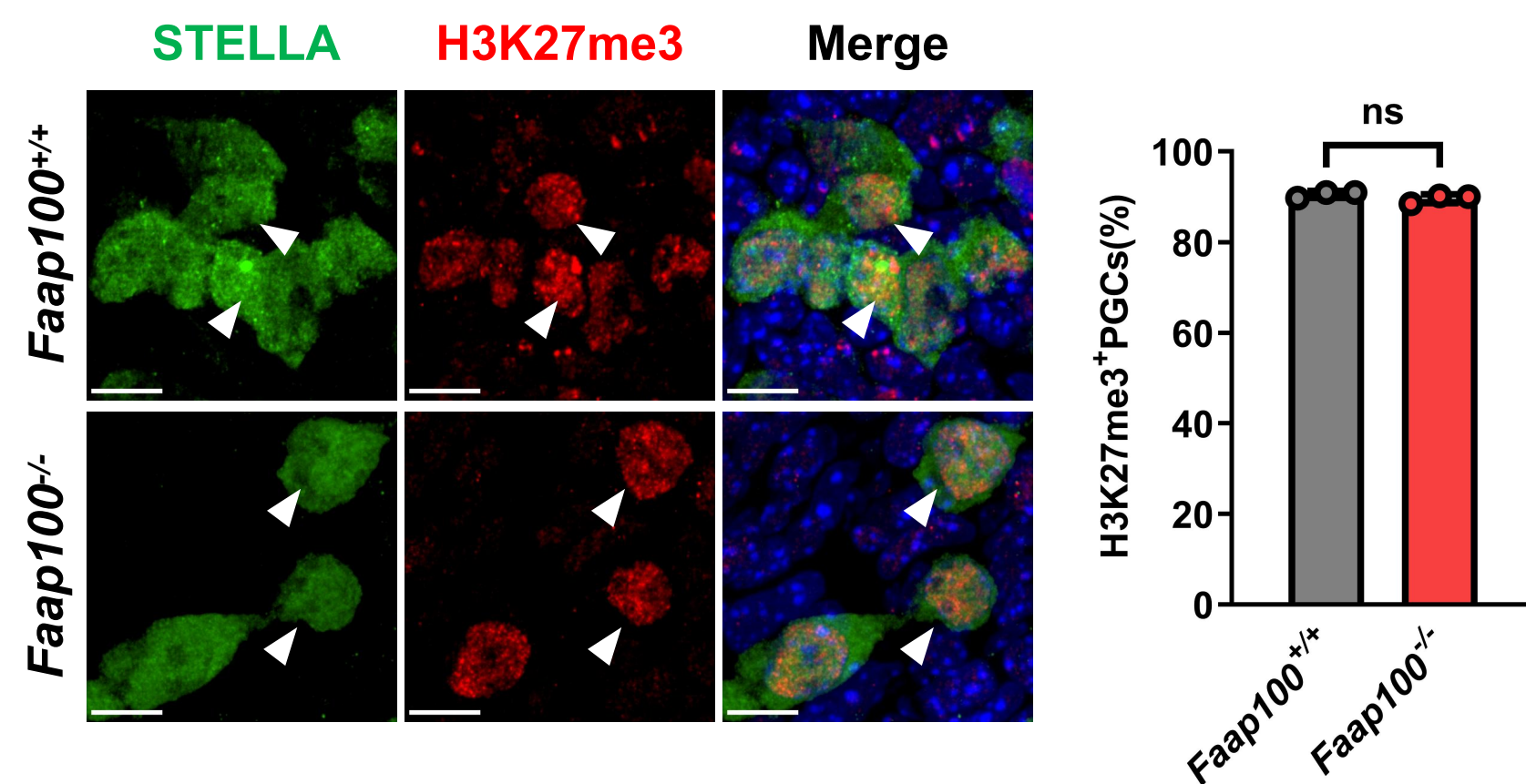

Supplement: Supplementary file 3 — Additional file 3: Fig. S3. No significant abnormalities of transcriptional activation and epigenetic modification were observed in Faap100-/- PGCs. A, Representative images and quantification of pSer5 Pol II signal intensity in E11.5 PGCs (STELLA+). n= 3/3 embryos (171/208/178; 150/172/150 PGCs). Scale bars, 10 μm. B, Representative images and quantification of pSer2 Pol II signal intensity in E11.5 PGCs (STELLA+). n= 3/3 embryos (203/161/185; 161/191/152 PGCs). Scale bars, 10 μm. C, Representative images and percentage of PGCs (STELLA+) stained negative for 5mC in E11.5 genital ridges. n= 3/3 embryos (201/214/206; 207/207/213 PGCs). Scale bars, 10 μm. D, Representative images and percentage of PGCs (STELLA+) stained negative for H3K9me2 in E11.5 genital ridges. n= 3/3 embryos (236/250/153; 145/140/160 PGCs). Scale bars, 10 μm. E, Representative images and percentage of PGCs (STELLA+) stained positive for H3K27me3 in E11.5 genital ridges. n= 3/3 embryos (476/359/200; 296/104/111 PGCs). Scale bars, 10 μm. The arrowheads in A-E indicate representative PGCs. Data are shown as the mean ± SEM (A, B) or mean ± SD (C-E). Unpaired two-tailed Student’s t-test (A-D) and two-tailed Mann-Whitney U-test (E). ns, not significant. [file 12915_2023_1676_MOESM3_ESM.pdf]

**A**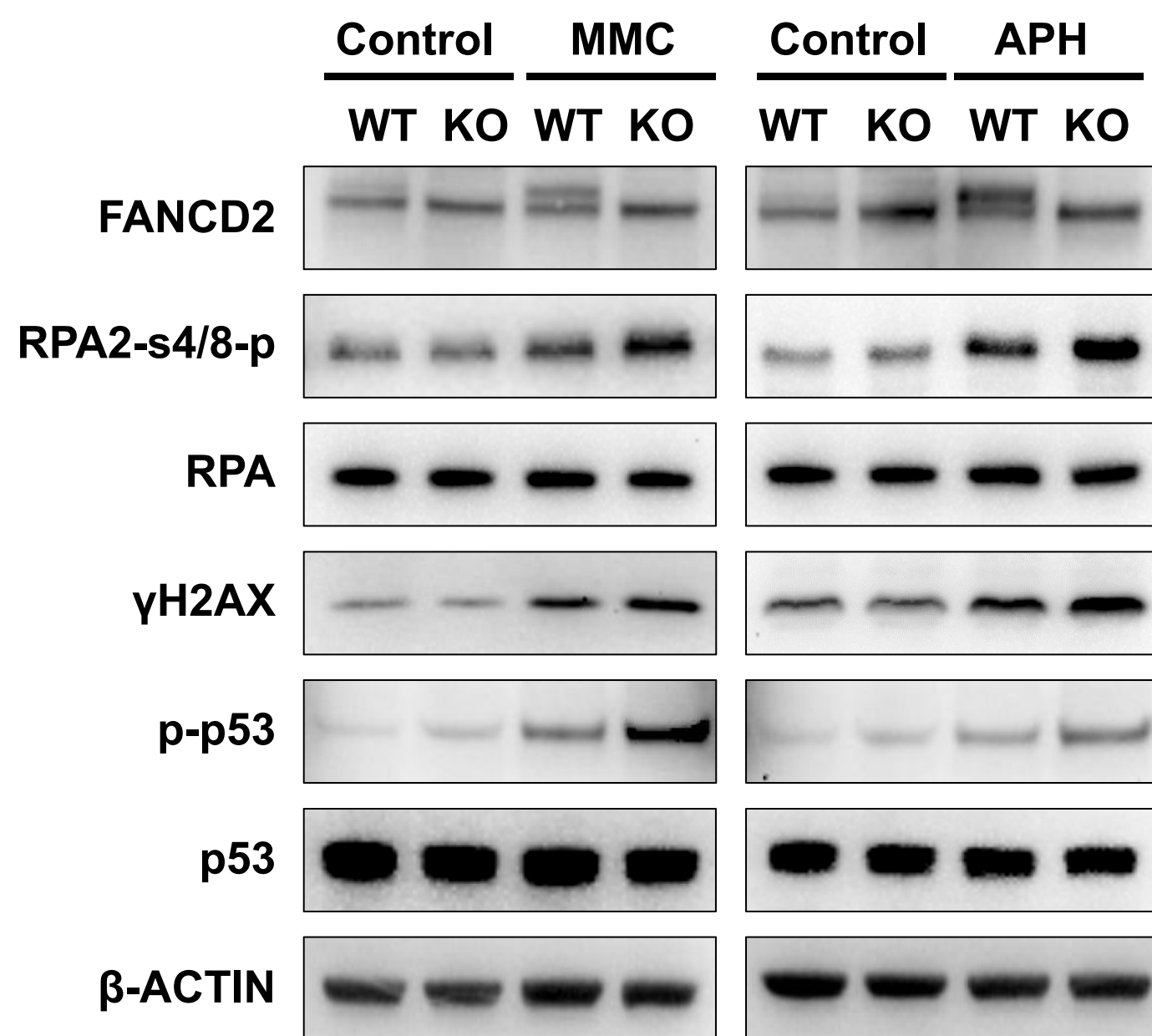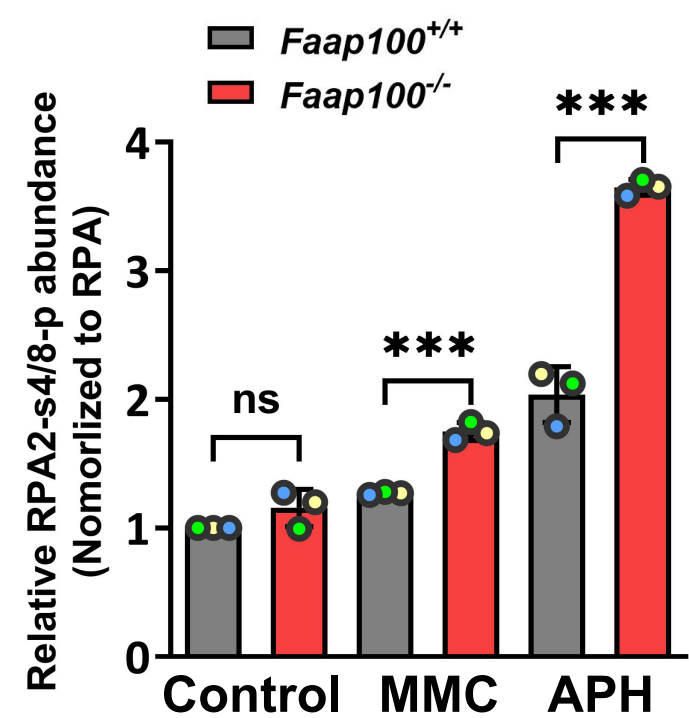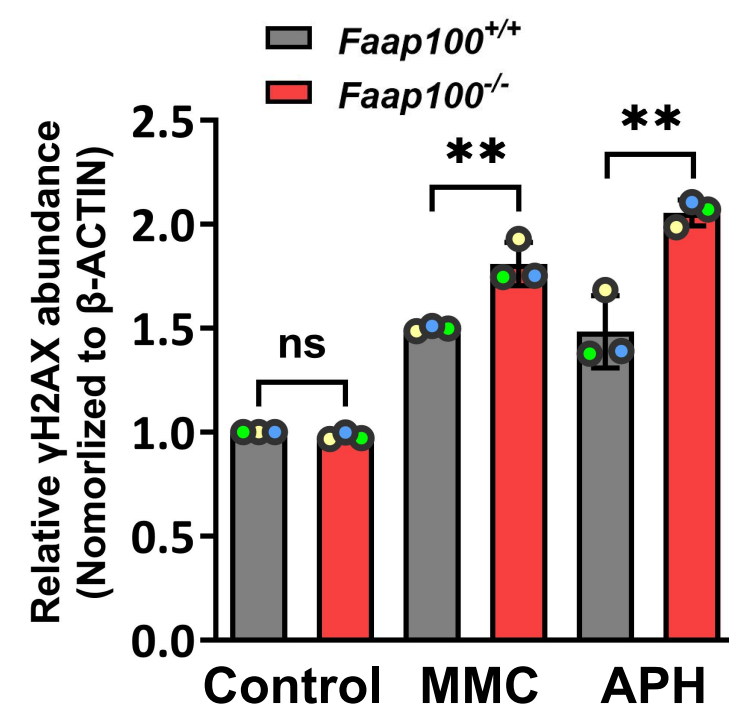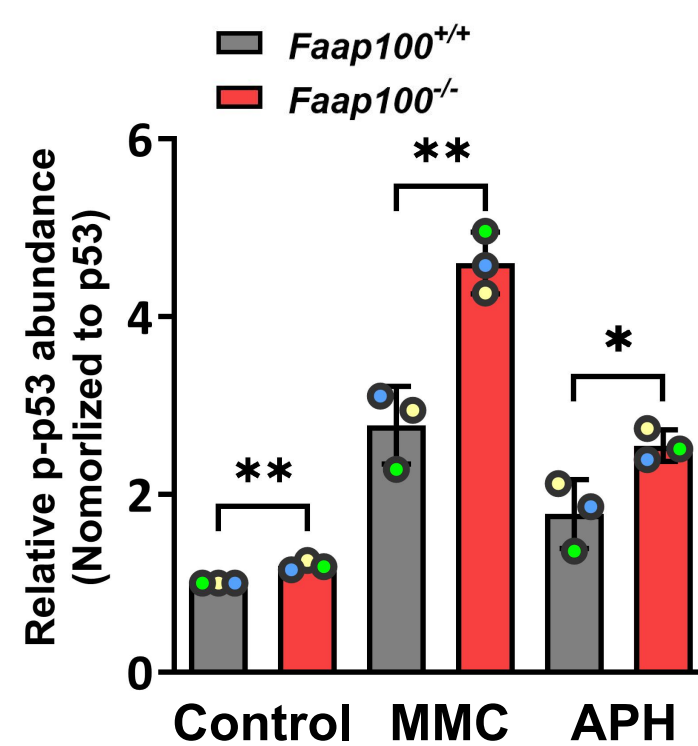**B**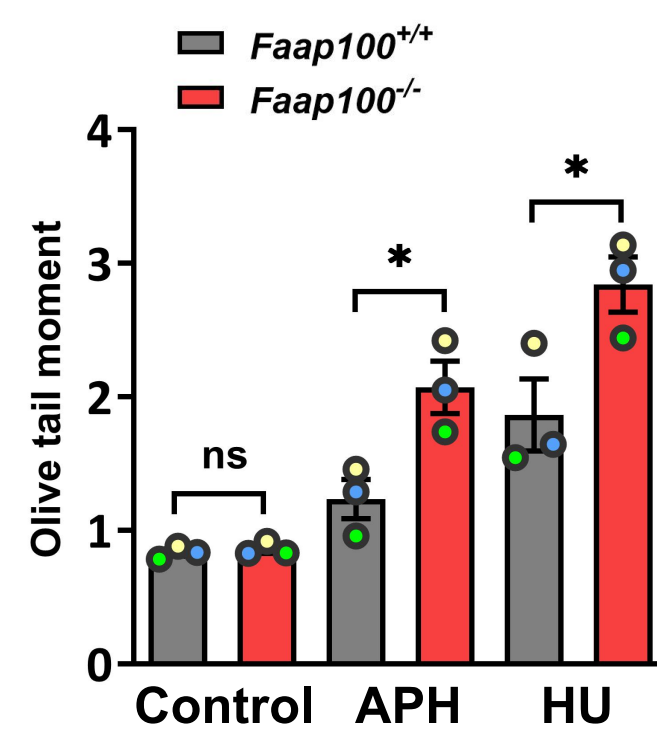**C**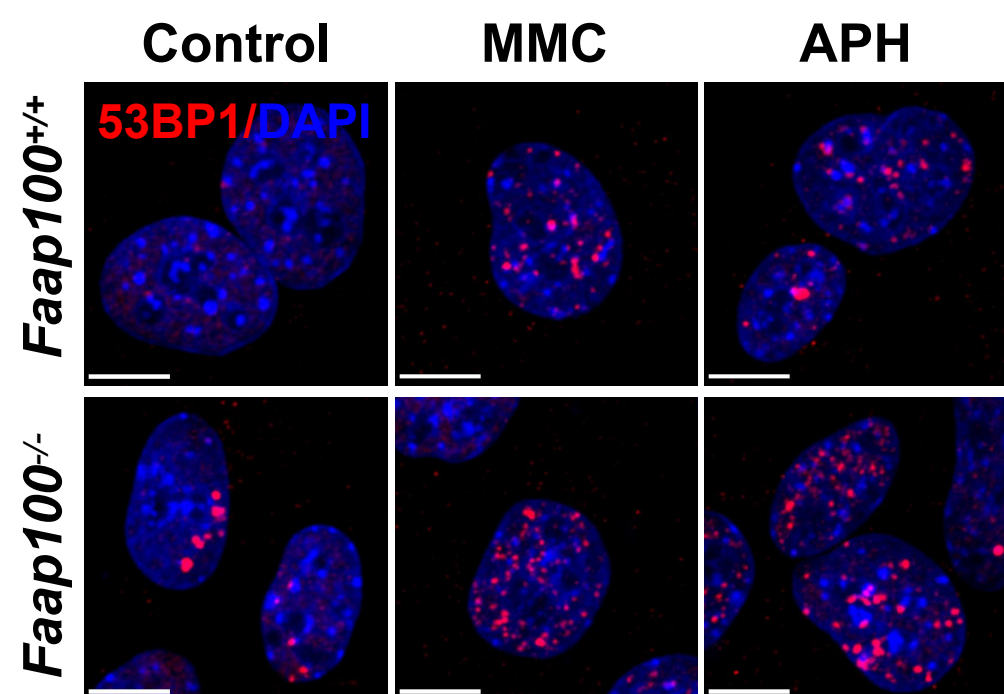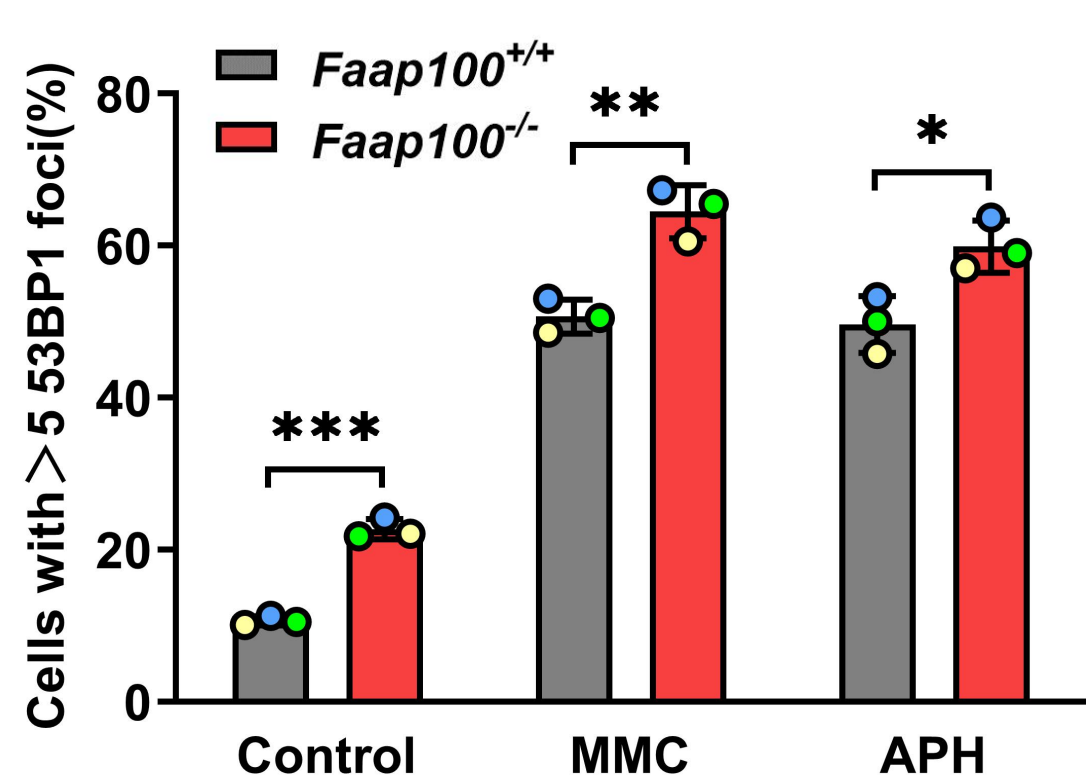**D**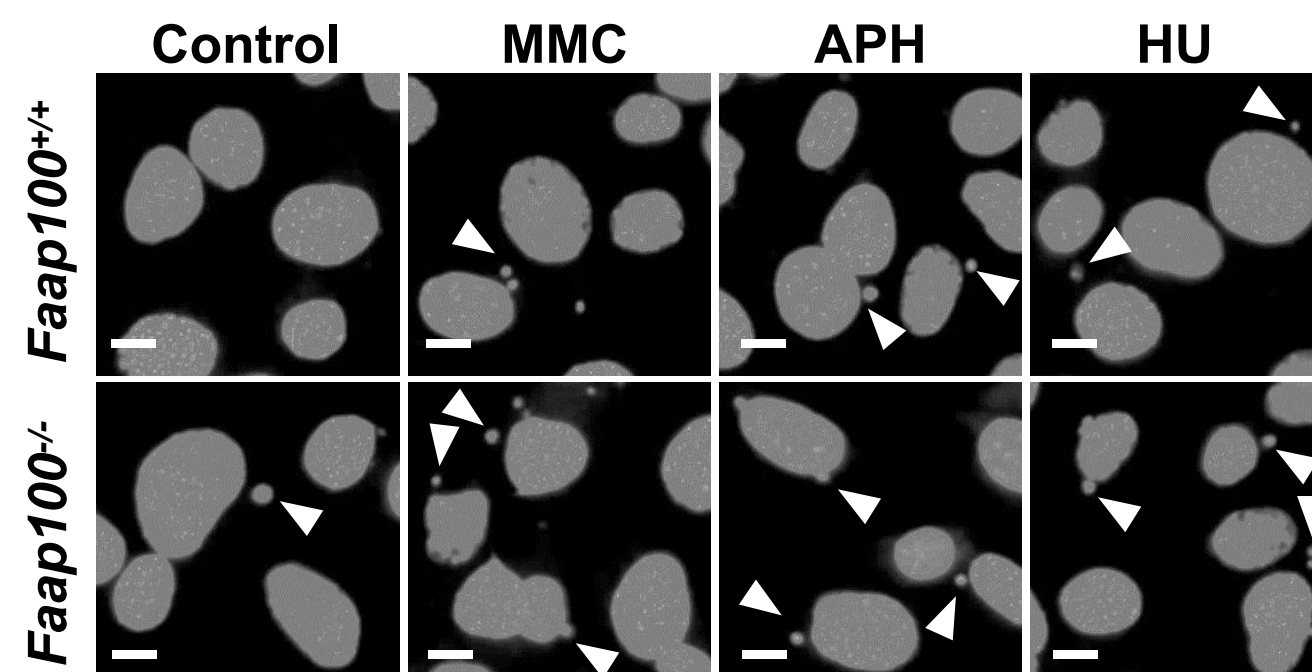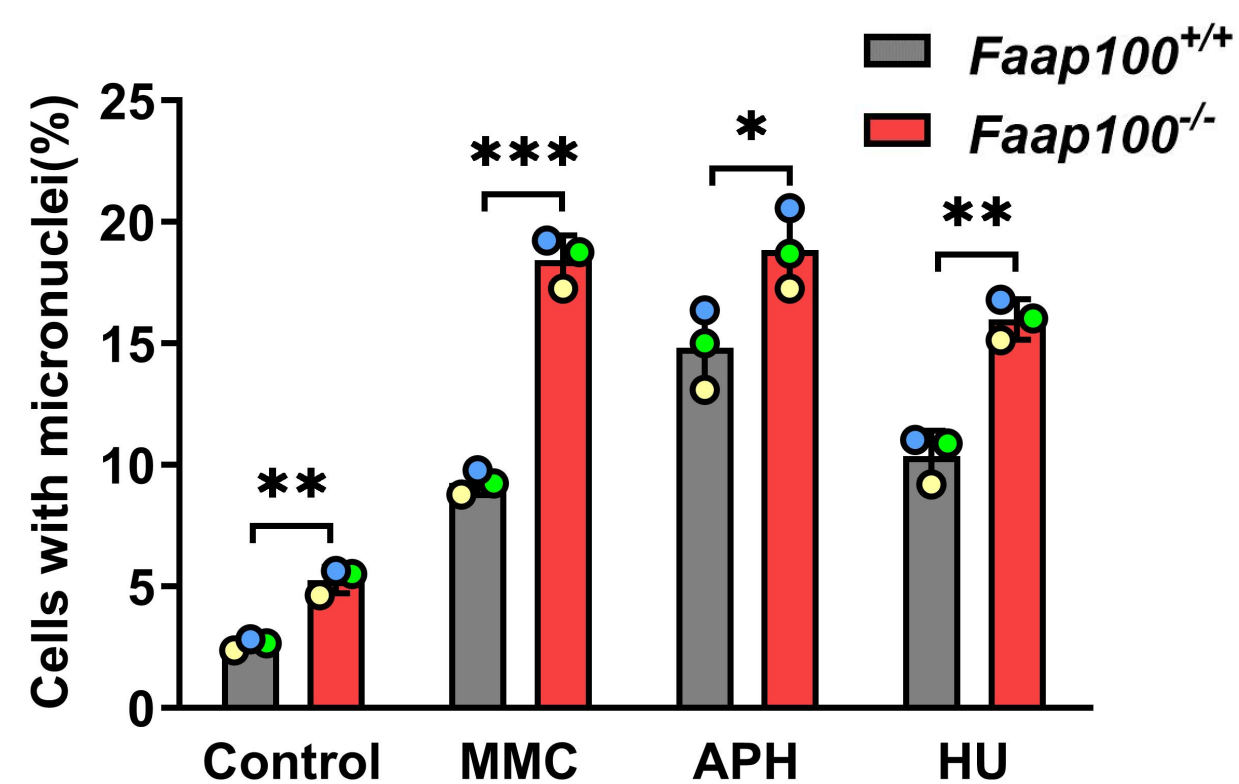

Supplement: Supplementary file 4 — Additional file 4: Fig. S4. FAAP100 loss causes elevated genome instability in MEFs. A, Representative images and quantification of the indicated proteins expression in whole-cell MEF extracts after exposure to 50 ng/ml MMC or 0.5 μM APH for 24 h. Three independent experiments were conducted. B, Quantification of the neutral comet assay tail moment in MEFs following exposure to 2 μM APH or 0.5 mM HU for 4 h. At least 200 cells were scored per group. Three independent experiments were conducted. C, Representative images and percentage of MEFs containing > 5 53BP1 foci following 100 ng/ml MMC or 2 μM APH treatment for 4 h. At least 200 cells were scored per group. Three independent experiments were conducted. Scale bars, 10 μm. D, Representative images and percentage of MEFs harbouring micronuclei after exposure to 50 ng/ml MMC, 0.5 μM APH or 0.5 mM HU for 24 h. At least 200 cells were scored per group. Three independent experiments were conducted. The arrowheads indicate micronuclei. Scale bars, 10 μm. Data are shown as the mean ± SD (A, C, D) or mean ± SEM (B). Unpaired two-tailed Student’s t-test (A-D), ns, not significant, *P < 0.05, **P < 0.01 and ***P < 0.001. [file 12915_2023_1676_MOESM4_ESM.pdf]

**Fig.S1C**

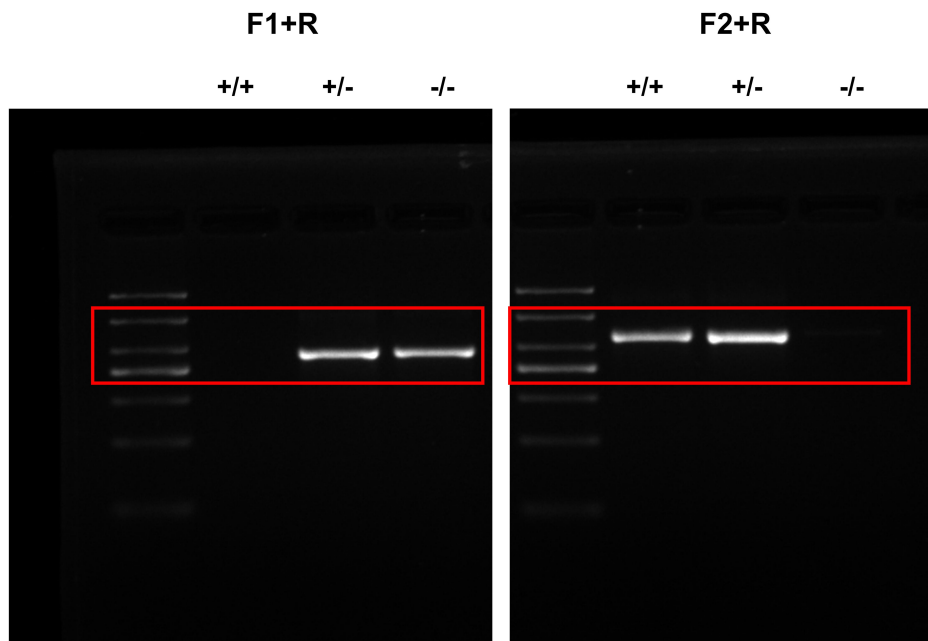

**Fig.S4A**

**FANCD2**

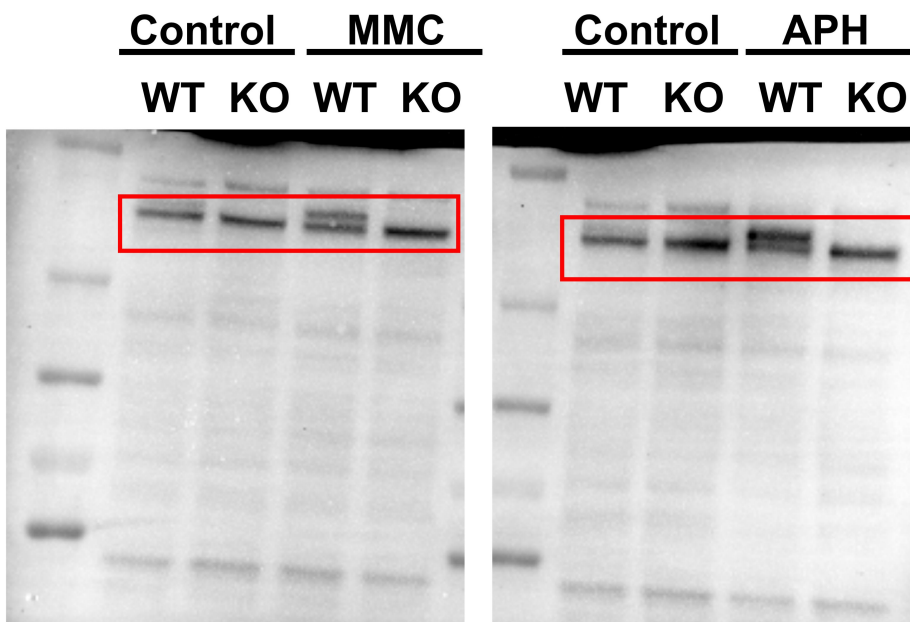

## RPA2-s4/8-p

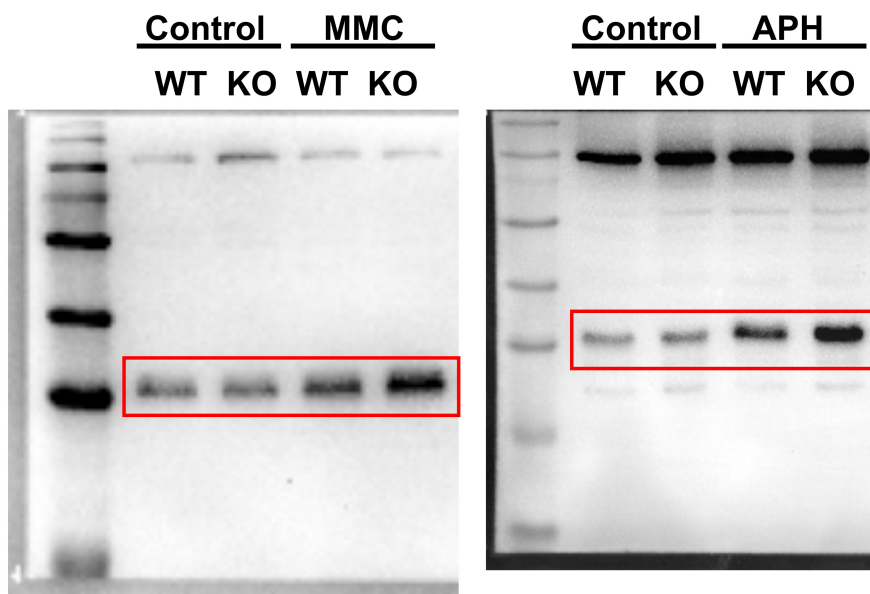

## RPA

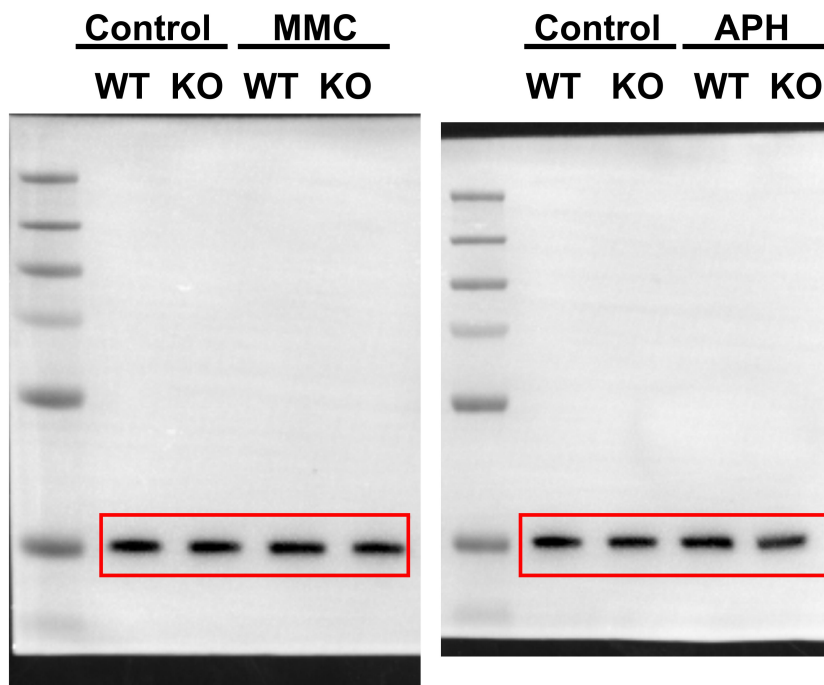

$\gamma$ H2AX

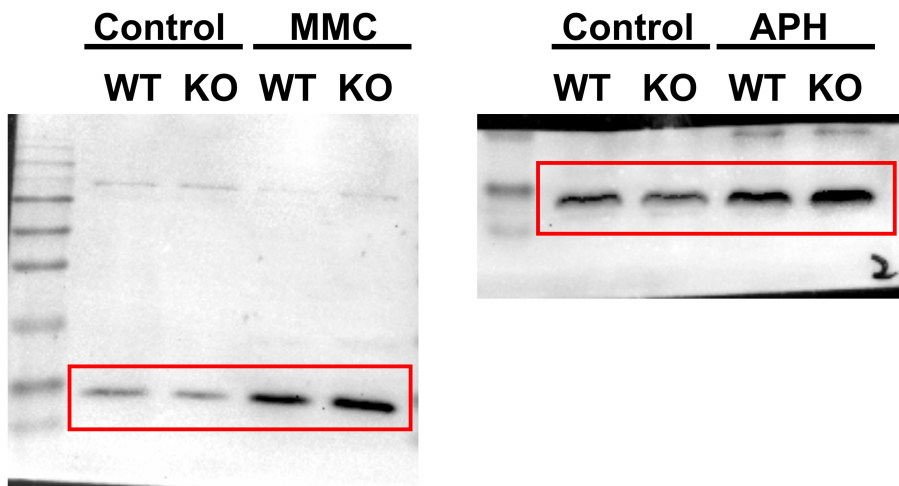

p-p53

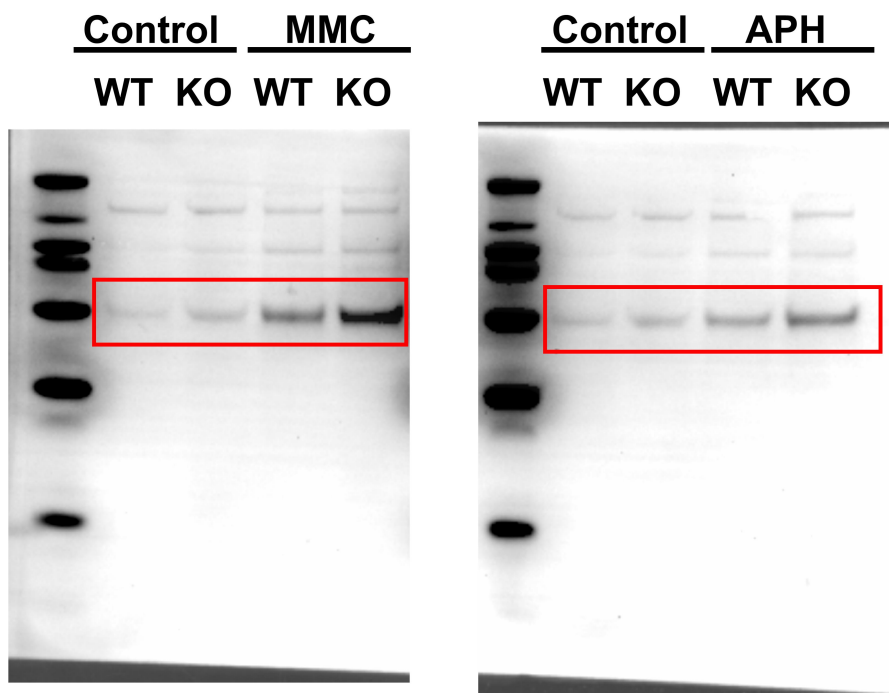

p53

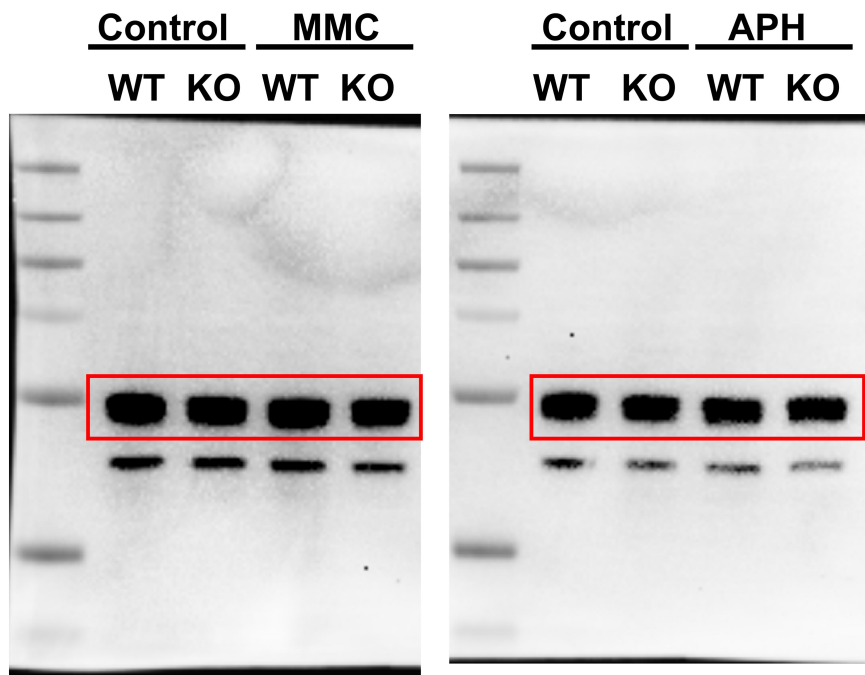

$\beta$ -ACTIN

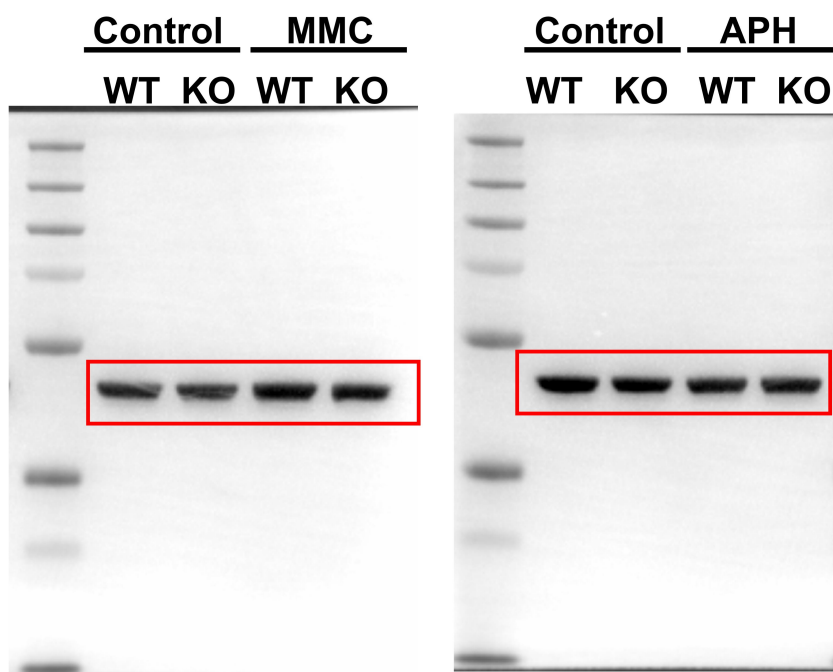

Supplement: Supplementary file 8 — Additional file 8. Uncropped gels/blots. [file 12915_2023_1676_MOESM8_ESM.pdf]
