# Supplementary material for: FAAP100 is required for the resolution of transcription-replication conflicts in primordial germ cells
Source: BMC Biol. 2023 Aug 15;21:174. doi: 10.1186/s12915-023-01676-1 (PMC10426154; doi:10.1186/s12915-023-01676-1)

A

Distribution of genotypes among offsprings of *Faap100*$^{+/-}$ intercross				
Groups	Total offsprings	Genotypes *P*=0.93		
		Faap100$^{+/+}$ N(%)	*Faap100*$^{+/-}$ N(%)	*Faap100*$^{-/-}$ N(%)
Observed	218	58(26.61)	107(49.08)	53(24.31)
Expected		54.5(25)	109(50)	54.5(25)

B

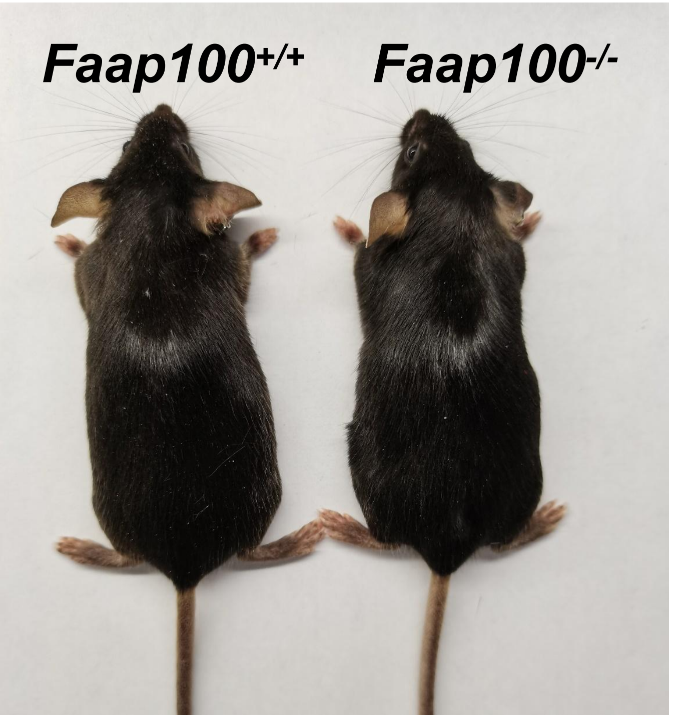

C

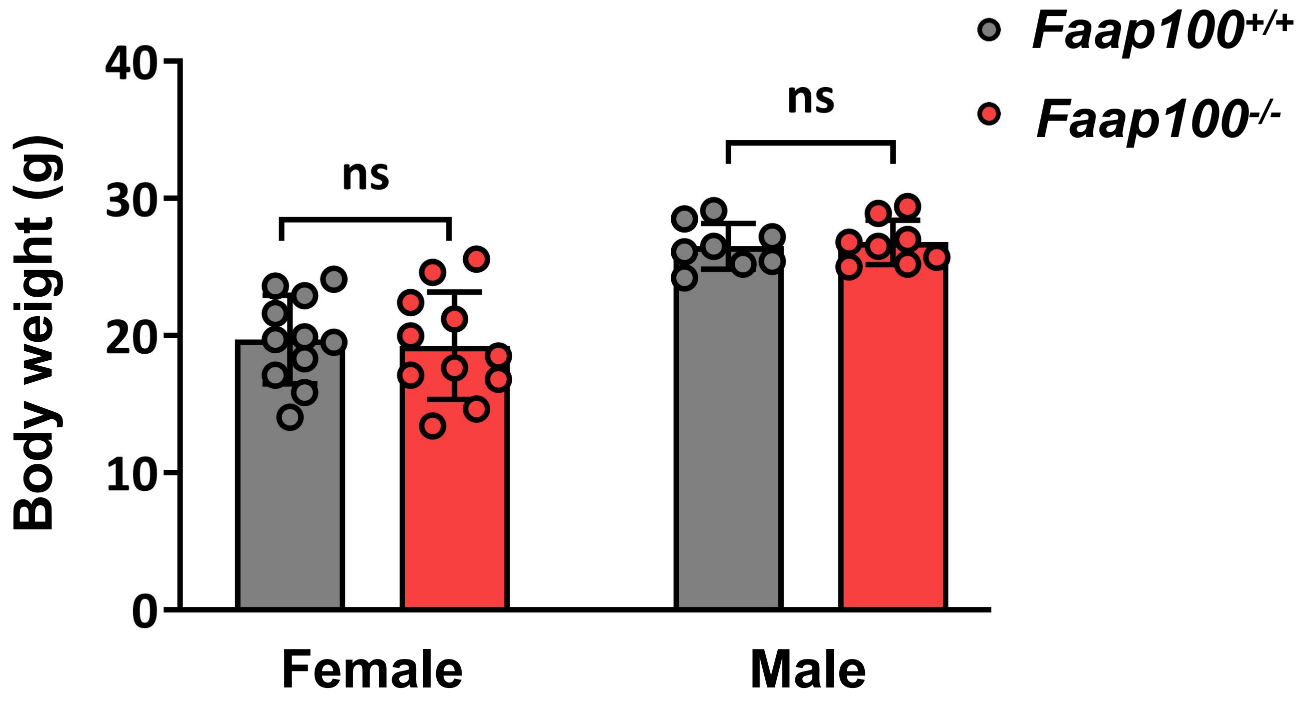

D

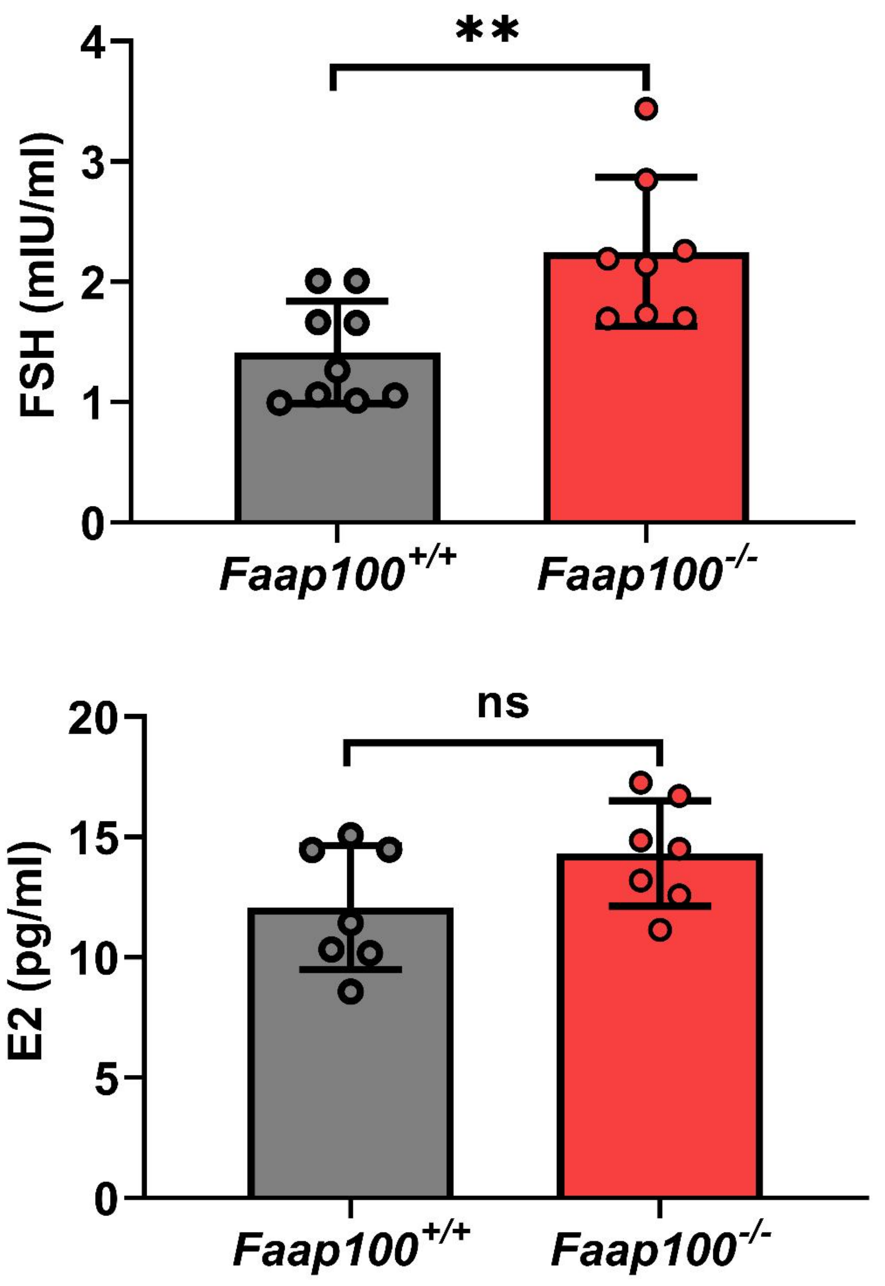

E

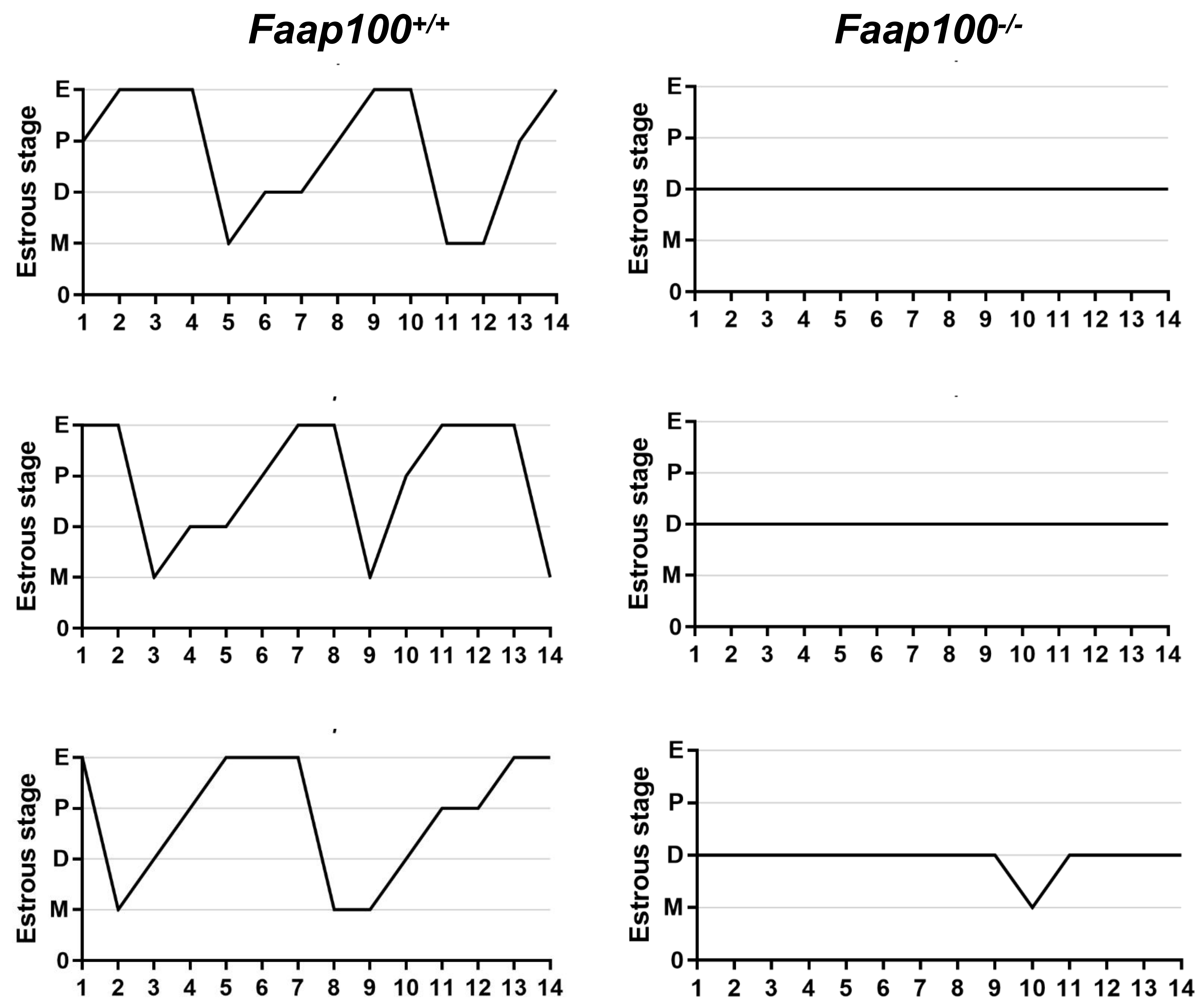

Supplement: Supplementary file 2 — Additional file 2: Fig. S2. Deletion of Faap100 results in premature ovarian insufficiency. A, Statistical analysis for genotyping results of pups resulting from the mating of heterozygous female mice with heterozygous male mice. n=218 pups. B, A representative photograph of 3-month-old wild-type and Faap100-/- female mice. C, Body weights of 3-month-old wild-type and Faap100-/- mice. n=11/11/8/8. D, Levels of FSH and E2 in the serum of 3-month-old wild-type and Faap100-/- female mice. FSH, n=9/8; E2, n=7/7. E, Estrous cycles of 3-month-old wild-type and Faap100-/- female mice. n=6/6. Representative data from 3 independent mice per genotype. E, estrus; P, proestrus; D, diestrus; M, metestrus. Data are shown as the mean ± SD (C, D). Chi-square test (A) and unpaired two-tailed Student’s t-test (C, D), ns, not significant and **P < 0.01. [file 12915_2023_1676_MOESM2_ESM.pdf]
