# Supplementary material for: FAAP100 is required for the resolution of transcription-replication conflicts in primordial germ cells
Source: BMC Biol. 2023 Aug 15;21:174. doi: 10.1186/s12915-023-01676-1 (PMC10426154; doi:10.1186/s12915-023-01676-1)
Supplement: Supplementary file 5 — Additional file 5: Table S1. Sequences of PCR primers used for genotyping. [file 12915_2023_1676_MOESM5_ESM.pdf]

Table S1. Sequences of PCR primers used for genotyping.

Primer	Sequence (5’-3’)
Faap100-F1	TGTGAGAGGTTTGTCCCAGTAGTAG
Faap100-F2	GTTGGACCACCAAAGGACGATG
Faap100-R	GACAACAGTATCAGGATGACAGGC
p53-F1	GCTTTCCCACCCTCGCATAAG
p53-F2	CCATAAGACAGGTGCTCCTCCAC
p53-R	TTCACTACAAAGGCTGAGCTGGAG
