# Supplementary material for: FAAP100 is required for the resolution of transcription-replication conflicts in primordial germ cells
Source: BMC Biol. 2023 Aug 15;21:174. doi: 10.1186/s12915-023-01676-1 (PMC10426154; doi:10.1186/s12915-023-01676-1)
Supplement: Supplementary file 6 — Additional file 6: Table S2. Antibodies. [file 12915_2023_1676_MOESM6_ESM.pdf]

Table S2. Antibodies.

Antibody (clone)	Catalog number	Company	Dilution rate
STELLA (Polyclonal)	AF2566	R&D systems	1:40 (IF)
DDX4 (mAbcam27591)	ab27591	Abcam	1:200(IF)<br>1: 5000 (IHC)
FOXL2 (Polyclonal)	ab5096	Abcam	1:200 (IF)
SOX9 (Polyclonal)	AB5535	Millipore	1:200 (IF)
Cleaved PARP1 (D6X6X)	94885	Cell signaling technology	1:200 (IF)
Ki67 (Polyclonal)	ab15580	Abcam	1:300 (IF)
Cyclin B1 (Polyclonal)	4138	Cell signaling technology	1:200 (IF)
pSer5 Pol II (EPR19015)	ab193467	Abcam	1:100 (IF)
pSer2 Pol II (EPR18855)	ab193468	Abcam	1:200 (IF)
5mC (RM231)	ab214727	Abcam	1:500 (IF)
H3K9me2 (Polyclonal)	07-441	Millipore	1:500 (IF)
H3K27me3 (Polyclonal)	07-449	Millipore	1:500 (IF)
FANCD2 (EPR2302)	ab108928	Abcam	1:300 (IF)<br>1:1000 (WB)
53BP1 (Polyclonal)	NB100-304	Novus biologicals	1:500 (IF)
p-p53 (D4S1H)	12571	Cell signaling technology	1:100 (IF)<br>1:1000 (WB)
RPA2 (EPR2877Y)	ab76420	Abcam	1:1000 (WB)
RPA2-S4/8-p (Polyclonal)	A300-245A	Bethyl	1:5000 (WB)
γH2AX (JBW301)	05-636	Millipore	1:1000 (WB)
p53 (2H9O)	32532s	Cell signaling technology	1:1000 (WB)
β-ACTIN (2D4H5)	66009-1-lg	Proteintech	1:3000 (WB)
Pol II (8WG16)	ab817	Abcam	1:100 (IF)
Pol II (Polyclonal)	ab26721	Abcam	1:200 (IF)
PCNA (PC10)	SC-56	Santa cruz biotechnology	1:50 (IF)
PCNA (D3H8P)	13110	Cell signaling technology	1:500 (IF)
S9.6 (Polyclonal)	Kf-Ab01137-23.0	Kerafast	1:200 (IF)
Fibrillarin (38F3)	Ab4566	Abcam	1:500 (IF)
BrdU (BU1/75 (ICR1))	Ab6326	Abcam	1:200 (IF)
BrdU (B44)	347580	BD Biosciences	1:50 (IF)
Donkey anti-Goat Alexa Fluor Plus 488 (polyclonal)	A32814	Invitrogen	1:500 (IF)
Donkey anti-Goat Alexa Fluor Plus 555 (polyclonal)	A32816	Invitrogen	1:500 (IF)

Donkey anti-Rabbit Alexa Fluor Plus 555 (polyclonal)	A32794	Invitrogen	1:500 (IF)
Donkey anti-Rabbit Alexa Fluor Plus 647 (polyclonal)	A32795	Invitrogen	1:500 (IF)
Donkey anti-Mouse Alexa Fluor Plus 488 (polyclonal)	A32766	Invitrogen	1:500 (IF)
Donkey anti-Mouse Alexa Fluor Plus 555 (polyclonal)	A31570	Invitrogen	1:500 (IF)
Donkey anti-Rat Alexa Fluor Plus 488 (polyclonal)	A48269	Invitrogen	1:500 (IF)
