# Supplementary material for: FAAP100 is required for the resolution of transcription-replication conflicts in primordial germ cells
Source: BMC Biol. 2023 Aug 15;21:174. doi: 10.1186/s12915-023-01676-1 (PMC10426154; doi:10.1186/s12915-023-01676-1)
Supplement: Supplementary file 7 — Additional file 7: Table S3. Reagents for assays. [file 12915_2023_1676_MOESM7_ESM.pdf]

Table S3. Reagents for assays.

Reagent	Catalog number	Company
RNAscope® 2.5 HD Reagent Kit-RED	322350	Advanced cell diagnostics
Duolink insitu PLA probe anti-rabbit minus	DUO92005	Sigma
Duolink insitu PLA probe anti-mouse plus	DUO92001	Sigma
Duolink insitu detection reagents red	DUO92008	Sigma
Cell-Light EdU Apollo643 In Vitro Kit	C10310-2	Ribobio
KAPA Mouse Genotyping Kit	KK7351	Sigma
1-Naphthyl phosphate, disodium salt	N7255	Sigma
Fast Red TR salt	F6760	Sigma
5-ethynyl-2'-deoxyuridine (EdU)	900584	Sigma
Mitomycin C (MMC)	M2320	TCI
Aphidicolin (APH)	504744	Sigma
Hydroxyuria (HU)	H8627	Sigma
5-iodo-2'-deoxyuridine (IdU)	I7125	Sigma
5-Chloro-2'-deoxy-uridine (CldU)	C6891	Sigma
